# Gray and white matter abnormality in patients with T2DM-related cognitive dysfunction: a systemic review and meta-analysis

**DOI:** 10.1038/s41387-022-00214-2

**Published:** 2022-08-15

**Authors:** Teng Ma, Ze-Yang Li, Ying Yu, Bo Hu, Yu Han, Min-Hua Ni, Yu-Xiang Huang, Hao-Han Chen, Wen Wang, Lin-Feng Yan, Guang-Bin Cui

**Affiliations:** 1 Department of Radiology, Functional and Molecular Imaging Key Lab of Shaanxi Province, Tangdu Hospital, Fourth Military Medical University (Air Force Medical University), 569 Xinsi Road, Xi’an, 710038 Shaanxi China; 2 Student Brigade, Fourth Military Medical University (Air Force Medical University), 169 Changle Road, Xi’an, 710032 Shaanxi China

**Keywords:** Type 2 diabetes, Cognitive neuroscience

## Abstract

**Aims/hypothesis:**

Brain structure abnormality in patients with type 2 diabetes mellitus (T2DM)-related cognitive dysfunction (T2DM-CD) has been reported for decades in magnetic resonance imaging (MRI) studies. However, the reliable results were still unclear. This study aimed to make a systemic review and meta-analysis to find the significant and consistent gray matter (GM) and white matter (WM) alterations in patients with T2DM-CD by comparing with the healthy controls (HCs).

**Methods:**

Published studies were systemically searched from PubMed, MEDLINE, Cochrane Library and Web of Science databases updated to November 14, 2021. Studies reporting abnormal GM or WM between patients with T2DM-CD and HCs were selected, and their significant peak coordinates (*x, y, z*) and effect sizes (*z*-score or *t*-value) were extracted to perform a voxel-based meta-analysis by anisotropic effect size-signed differential mapping (AES-SDM) 5.15 software.

**Results:**

Total 15 studies and 16 datasets (1550 participants) from 7531 results were involved in this study. Compared to HCs, patients with T2DM-CD showed significant and consistent decreased GM in right superior frontal gyrus, medial orbital (PFCventmed. R, BA 11), left superior temporal gyrus (STG. L, BA 48), and right calcarine fissure / surrounding cortex (CAL. R, BA 17), as well as decreased fractional anisotropy (FA) in right inferior network, inferior fronto-occipital fasciculus (IFOF. R), right inferior network, longitudinal fasciculus (ILF. R), and undefined area (32, −60, −42) of cerebellum. Meta-regression showed the positive relationship between decreased GM in PFCventmed.R and MoCA score, the positive relationship between decreased GM in STG.L and BMI, as well as the positive relationship between the decreased FA in IFOF.R and age or BMI.

**Conclusions/interpretation:**

T2DM impairs the cognitive function by affecting the specific brain structures. GM atrophy in PFCventmed. R (BA 11), STG. L (BA 48), and CAL. R (BA 17), as well as WM injury in IFOF. R, ILF. R, and undefined area (32, −60, −42) of cerebellum. And those brain regions may be valuable targets for future researches. Age, BMI, and MoCA score have a potential influence on the altered GM or WM in T2DM-CD.

## Introduction

Type 2 diabetes mellitus (T2DM) has been widely regarded as a common challenge in human health. It was estimated that ~36% of the population in Asia suffered from T2DM, and the patients with T2DM in America could be double in 2050 [1, 2]. Unfortunately, T2DM is also the leading cause of a range of complications (e.g., stroke, cognitive dysfunction, and depression), which will further place a heavy burden on human health [3, 4]. Cognitive impairment is one of the most common complications of T2DM [5]. Previous study reported that a 150% increased risk of cognitive impairment in human with T2DM [6]. However, there was still a lack of reliable biomarkers in clinical practice, making early prediction and treatment of T2DM-CD difficult [7]. Further exploration of the underlying mechanism of T2DM-CD was of great significance for clinical intervention strategies and therapeutic effects [6, 8]. Recently, more and more studies have begun to focus on the potential neural mechanisms in T2DM-CD. Neuroimaging is one of the most important techniques for exploring T2DM-CD. Gray matter (GM) and white matter (WM) are two sensitive markers in neuroimaging studies, which indicate the changes in brain structure caused by T2DM [9–12].

Voxel-based morphometry (VBM) and surface-based morphometry (SBM) are two very important methods in studies exploring changes in brain GM [13]. Previous SBM study reported that patients with T2DM-CD showed the cortical/subcortical GM atrophy in thalamus, putamen, and hippocampus compared to healthy controls (HCs) [14]. Besides, it was reported that patients with T2DM-CD showed the lesser gray matter volume (GMV) in the right temporal and left occipital cortex than HCs in VBM studies [15]. In addition, study found that patients with T2DM-CD had the lower cortical thickness in temporal, parietal, frontal, and occipital regions than HCs [16]. Diffusion tensor imaging (DTI) is one of the most important techniques to explore the brain WM alterations in T2DM. The voxel-based analysis (VBA) and tract-based spatial statistics (TBSS) are two major methods based on DTI to explore the integrity of WM, the injury degree of which can be indicated by the fractional anisotropy (FA) and the mean diffusivity (MD) [17]. According to the TBSS study, patients with T2DM-CD showed the injured WM in corpus callosum (CC), left anterior limb of the internal and external capsule compared to HCs [18]. However, patients with T2DM-related amnesic mild cognitive impairment (T2DM-aMCI) had the significant decreased FA in anterior thalamic radiation (ATR), corticospinal tract (CST), cingulum, inferior fronto-occipital fasciculus (IFOF), superior longitudinal fasciculus (SLF), uncinate fasciculus (UF), and left superior longitudinal fasciculus temporal part (SLF-temp) compared to HCs [19]. Taken together, studies above have demonstrated that the GM and WM abnormalities played a crucial role in the process of T2DM-CD.

However, those findings of brain structure abnormalities in T2DM-CD in previous neuroimaging studies were heterogenous. Although previous systemic review and meta-analysis studies have explored the altered GM and WM in T2DM, significant and consistent results in T2DM-CD were still lack. Therefore, a systemic review and meta-analysis of brain structural changes in T2DM-CD is warranted. In this study, we aimed to collect the studies on GM or WM alterations between T2DM-CD and HCs, as well as explore the characteristic brain regions that could provide a target for further researches. Before making this meta-analysis, we made two hypotheses: (1) GM and the adjacent WM in specific brain regions might be damaged simultaneously, (2) brain regions involved in cognitive regulation might be impaired.

## Methods

This systemic review and meta-analysis followed the PRISMA statement checklists [20], which was showed in Supplementary Table 1. The protocols were registered in PROSPERO (CRD42021248423; CRD42021249713) (https://www.crd.york.ac.uk/prospero/), which were showed in Supplementary Table 2. All works were performed by two reviewers (TM and ZYL) independently, any controversial questions were consulted and solved with the third reviewer (LFY).

### Systemic search and study selection

The systemic search of PubMed, MEDLINE, Cochrane Library and Web of Science Databases updated to November 14, 2021. Items used in this systemic search were showed as follows: (“diabetes mellitus, type 2” OR “type 2 diabetes” OR “NIDDM”, “T2DM” OR “diabetes mellitus, type II”) AND (“cognitive dysfunction”, “cognitive decline”, “cognitive impairment”, and “cognitive dissonance”) AND (“cerebral cortex”, “gray matter”, “VBM”, “voxel-based morphometry”, “cortical thickness”, “thickness”, “cortical thinning”, and “thinning”); (“diabetes mellitus, type 2” OR “type 2 diabetes” OR “NIDDM”, “T2DM” OR “diabetes mellitus, type II”) AND (“cognitive dysfunction”, “cognitive decline”, “cognitive impairment”, “cognitive dissonance”) AND (“white matter” OR “fractional anisotropy” OR “FA” OR “mean diffusivity” OR “MD” OR “VBA” OR “TBSS” OR “voxel-based analysis” OR “Tract-Based Spatial Statistics”) [21–23]. Detailed search strategies were showed in Supplementary Table 3. In addition, references to included studies and relevant reviews were searched as a supplement.

Inclusion criteria: (1) study reported the GM/WM abnormality in T2DM; (2) Between-group comparison of T2DM-CD and HCs; (3) study included male and female subjects (age > 18 years old); (4) reported available coordinates (*x*, *y*, *z*) in standard space (e.g., Montreal Neurological Institute (MNI) or Talairach (TAL)); (5) Cognitive assessments were performed in all subjects, and T2DM had lower cognitive scores than HCs. Exclusion criteria: (1) lacked HCs; (2) not adult; (3) single gender; (4) regions-of-interest (ROI) studies; (5) lack of cognitive assessment; (6) animal studies; (7) not original studies; (8) not gray/white matter studies; (9) lack coordinate information; (10) not TBSS or VBA method.

### Quality assessment, data extraction, and conversion

The quality of total 15 studies (11 GM datasets and 5 WM datasets) included was assessed by 12-point checklists, and the total score of which more than 6 was regarded as available [24, 25].

The quality assessment information of the studies included was detailed in Supplementary Table 4. General information (researchers, cognitive scales, age, disease duration, BMI, HbA1c, *P* value, and standard space) of all studies included were showed in Table 1. The detailed cognitive scores were showed in Supplementary Table 5. Significant peak coordinates (*x*, *y*, *z*) and effect sizes (*t* value or *z* score) of GM/WM abnormality between T2DM-CD and HCs were collected for meta-analysis. The data conversion between *t*-values and z-scores was performed on the SDM website (https://www.sdmproject.com/).

**Table 1:** General information of 15 studies included (gray and white matter).

Study	Participants (female)			Neuropsychology examinations	Age means (sd.)		*P* value	Disease duration (year)	Field strength (T)	T2DM HbA1c (%)	T2DM BMI (kg/m^2^)	Coordinate space	Subject
	Total	T2DM	HC		T2DM	HC							
Moran et al. [39]	713 (308)	350 (140)	363 (168)	Other	67.8 (6.90)	72.1 (7.20)	0.001 (corrected)	NA	1.5	7.20	31.30	NO	Gray matter
Li et al. [14]	56 (35)	28 (15)	28 (20)	MoCA + MMSE	56.5 (1.10)	53.9 (1.20)	0.05 (corrected)	8.20	3.0	9.80	24.80	MNI	Gray matter
Chen et al. [35]	22 (16)	11(8)	11(8)	MMSE	61.2 (NA)	56.2 (NA)	0.05 (corrected)	NA	3.0	8.30	25.40	MNI	Gray matter
Natalia et al. [42]	50 (19)	25 (8)	25 (11)	MMSE + Other	60.0 (4.60)	57.8 (5.40)	0.001 (uncorrected)	NA	3.0	6.67	28.60	TAL	Gray matter
Wang et al. [11]	46 (16)	23 (7)	23 (9)	MoCA + MMSE + Other	53.1 (9.60)	53.9 (9.20)	0.001 (uncorrected)	7.00	3.0	8.30	25.90	MNI	Gray matter
Yoon et al. [16^a^]	150 (75)	100 (50)	50 (25)	Other	49.2 (7.70)	49.0 (7.80)	0.05 (corrected)	1.83	1.5	7.12	25.50	TAL	Gray matter
Zhang et al. [37]	57 (37)	29 (17)	28 (20)	MoCA + MMSE	56.21 (7.69)	52.24 (9.09)	0.05 (corrected)	8.18	3.0	7.78	24.35	MNI	Gray matter
Zhou et al. [38]	40 (20)	21 (11)	19 (9)	MMSE + Other	68 (5.00)	69 (5.00)	0.001 (uncorrected)	4.70	1.5	8.60	25.70	TAL	Gray matter
Wei et al. [25]	63 (30)	31 (15)	32 (15)	MMSE	58.42 (8.42)	55.03 (7.81)	0.001 (uncorrected)	5.73	1.5	6.77	26.30	MNI	Gray matter
Duan et al. [36]	104 (44)	52 (18)	52 (26)	MoCA + MMSE + Other	54.35 (5.33)	53.31 (4.30)	0.05 (corrected)	9.19	3.0	8.42	24.32	MNI	Gray matter
Feng et al. [40]	40 (NA)	20 (NA)	20 (NA)	MoCA + Other	36.45 (3.72)	34.05 (4.78)	0.05 (corrected)	3.79	3.0	9.80	25.41	MNI	Gray matter
Cui et al. [46]	80 (45)	38 (18)	42 (27)	MMSE + Other	60.2 (7.4)	58.2 (6.4)	0.05 (corrected)	9.0	3.0	8.00	24.90	MNI	White matter
Kim et al. [43]	40 (22)	20 (11)	20 (11)	Other	54.6 (2.3)	54.3 (2.4)	0.05 (corrected)	12.1	3.0	10.70	24.70	MNI	White matter
Xiong et al. [44]	48 (30)	20 (12)	28 (18)	MMSE + MoCA + Other	63.55 (5.81)	59.65 (5.98)	0.01 (corrected)	9.09	3.0	8.15	24.37	MNI	White matter
Yau et al. [45]	41 (20)	24 (11)	17 (9)	Other	57.21 (8.05)	56.44 (6.94)	0.01 (corrected)	7.94	1.5	7.83	32.13	TAL	White matter
Yoon et al. [16]^a^	150 (75)	100 (50)	50 (25)	Other	49.2 (7.70)	49.0 (7.80)	0.05 (corrected)	1.83	1.5	7.12	25.50	MNI	White matter

*MMSE* Mini-Mental State Examination, *MoCA* Montreal Cognitive Assessment, *Other* examinations other than the MMSE or MoCA, *TAL* Talairach, *MNI* Montreal Neurological Institute, *NO* Stereotaxic space, *NA* not available.

^a^The same study.

### Voxel-based meta-analysis by AES-SDM

The meta-analyses of GM and WM were respectively performed in the “Gray Matter” and “Fractional Anisotropy” templates of the anisotropic effect size-signed differential mapping (AES-SDM) 5.15 software [26, 27]. This meta-analysis followed the standard processes of AES-SDM that have been explanted in previous studies [28, 29], which were briefly described as follows: (1) By extracting the peak coordinates and effect sizes of GM/WM anomalies between T2DM-CD and HC, new statistical maps were recreated separately for each dataset in standard MNI map; (2) The mean map was created from the random-effects mean of the dataset maps, which was weighted by the sample size, intra-study variance, and between group heterogeneity; (3) a quantitative meta-analytic comparison of altered GM/WM was performed by calculating statistical differences in each voxel between patients with T2DM-CD and HCs; (4) the AES-SDM default parameters (Anisotropy = 1, Isotropic full width at half maximum (FWHM) = 20 mm, Probability = 0.005, Peak height threshold = 1, Extent threshold = 10 voxels) and 50-randomization test were adopted in this study [30]. The approach to pool the VBM and SBM studies into meta-analysis was shown in Supplementary Fig. 1 (https://www.nitrc.org/forum/forum.php?forum_id=3982). All significant results were visualized by MRIcroGL software [31, 32].

### Heterogeneity, sensitivity, and publication bias assessment

The heterogeneity between-study in main results was checked by the Q-test in SDM default, and the forest plots were visualized by R software 4.1.2 (https://www.r-project.org/). Whole-brain voxel-based jackknife sensitivity was performed to assess the reliability of results by repeating the same steps of main analysis and iteratively removing the included studies iteratively. It was regarded as significant and robust when the main results were reproducible for all or most of the analysis times [26]. The publication bias was checked by the Egger’s test in SDM default, which was visualized by funnel plots [33, 34].

### Meta-regression analysis

Meta-regression was performed to explore the possible effect of risk factors (age, percentage of female patients, disease duration, MMSE/MoCA score, BMI, and HbA1c) on GM/WM abnormalities in T2DM-CD. The linear model meta-regression and *P* = 0.0005 were selected in this study.

## Results

### Systemic search information and characteristics of included studies

**Fig. 1:**
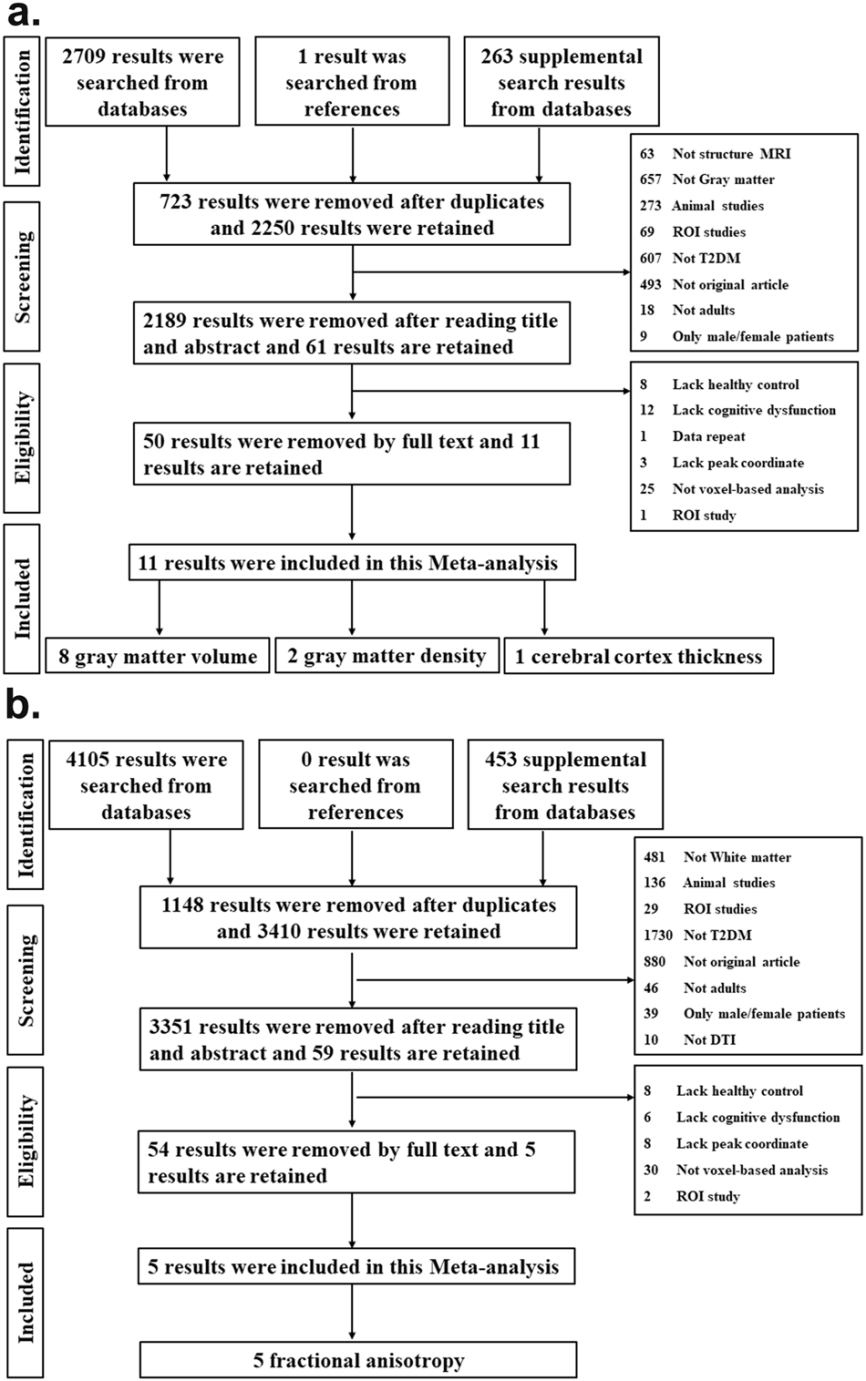
Search flow diagram followed the PRISMA statement. The systemic searches were separately performed on GM (**a**) and WM studies (**b**).

Total 7531 results were searched from 4 databases and relevant references. The 1871 results were further removed because of duplication. The remaining 5660 results were screened with the title and abstract, and 5540 results of which were removed. The full-text of remained 120 results were read, and 104 results of which were removed. In the end, total 15 studies (16 datasets) meeting the inclusion criteria were included in this study, which consisted of 11 GM datasets including 7 GMV [15, 35–40], 2 gray matter density (GMD) [40–42], and 2 cerebral cortical thickness [14, 16], as well, as 5 WM datasets including 5 FA abnormality [16, 43–46]. The detailed search flow diagram was showed in Fig. 1.

### GM abnormality between T2DM-CD and HCs

**Fig. 2:**
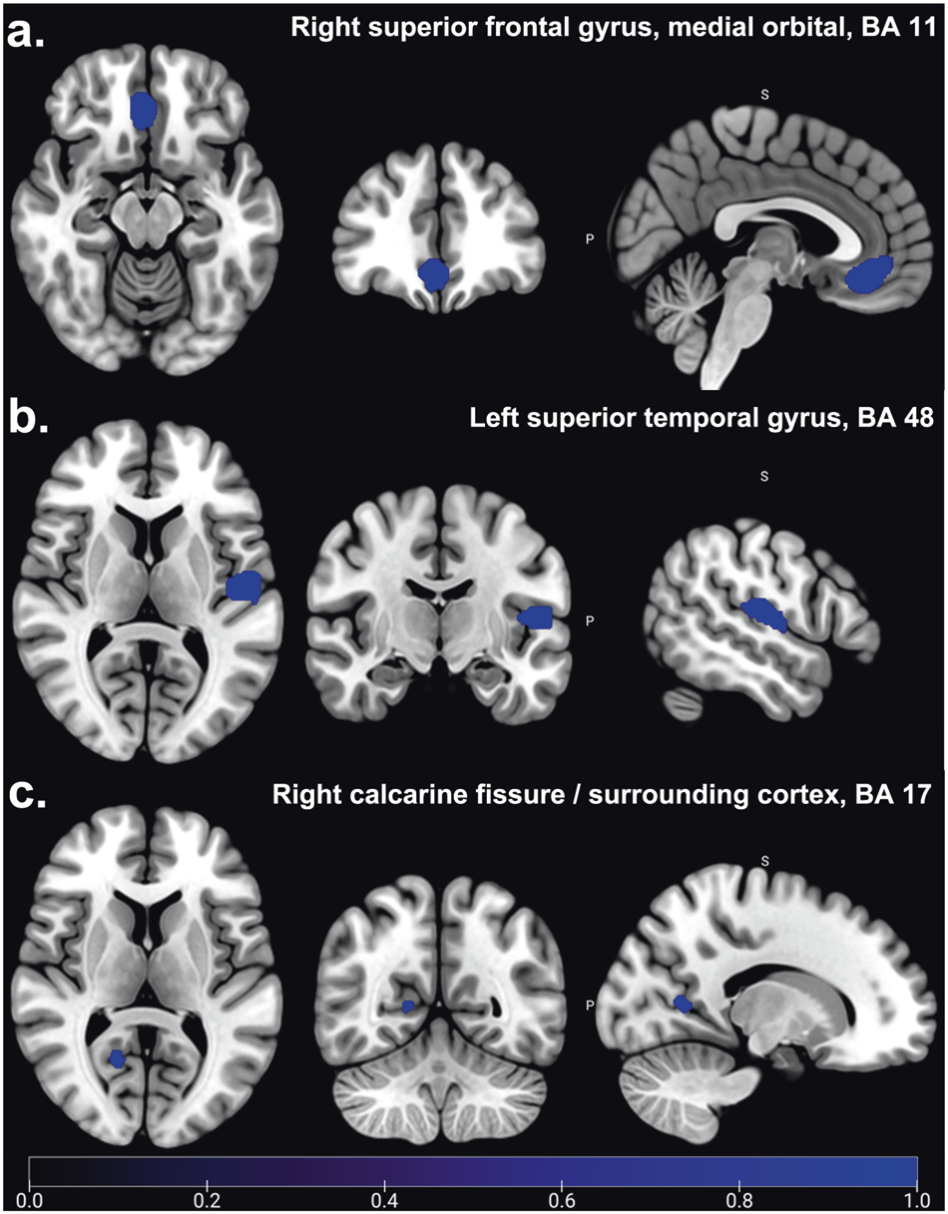
Significant GM abnormalities between T2DM-CD and HCs. Patients with T2DM-CD showed significant and consistent decreased GM (Blue) in right superior frontal gyrus, medial orbital (PFCventmed.R, BA 11) (**a**), left superior temporal gyrus (STG.L, BA 48) (**b**), and right calcarine fissure/surrounding cortex (CAL.R, BA 17) (**c**) compared to HCs (*P* < 0.005). No increased GM was found in T2DM-CD compared to HCs (*P* = 0.005).

According to the main analysis, patients with T2DM-CD showed the significant and consistent GM reduction in right superior frontal gyrus, medial orbital (PFCventmed. R) (BA 11, *MNI*: 4, 38, −12; *SDM-Z* = −2.193, *P* < 0.001), left superior temporal gyrus (STG.L) (BA 48, *MNI*: −52, −16,12, *SDM-Z* = −2.174, *P* < 0.001), and right calcarine fissure/surrounding cortex (CAL.R) (BA 17, *MNI*: 16, −58,8, *SDM-Z* = −1.879, *P* < 0.005) compared to HCs (Table 2, Fig. 2). No increased GM was found in T2DM-CD compared to HCs. Q statistic showed the heterogeneity in main results (*Q* = 46.682, *P* < 0.000) (Supplementary Table 6, Supplementary Fig. 2). Jackknife analysis showed 10 of 11 times in PFCventmed. R, 8 of 11 times in STG. L, and 8 of 11 times in CAL. R (Supplementary Table 7). Egger’s test showed the publication bias in PFCventmed.R (*P* < 0.05), while no publication bias in in STG.L and CAL.R (*P* > 0.05) (Supplementary Fig. 3).

**Table 2:** Significant decreased GM and WM in patients with T2DM-CD compared with HCs.

MNI coordinate	*SDM-Z*	*P*	Voxels	Description	Jackknife
Gray matter					
4,38, −12	−2.193	<0.001	435	Right superior frontal gyrus, medial orbital, BA 11	10/11
−52, −16,12	−2.174	<0.001	419	Left superior temporal gyrus, BA 48	8/11
16, −58,8	−1.879	<0.005	51	Right calcarine fissure / surrounding cortex, BA 17	8/11
White matter					
36, −12, −12	−1.402	<0.001	57	Right inferior network, inferior fronto-occipital fasciculus	4/5
54, −18, −14	−1.038	<0.005	20	Right inferior network, inferior longitudinal fasciculus	4/5
32, −60, −42	−1.017	<0.005	15	Undefined	4/5

*BA* Brodmann area.

### WM abnormality between T2DM-CD and HCs

**Fig. 3:**
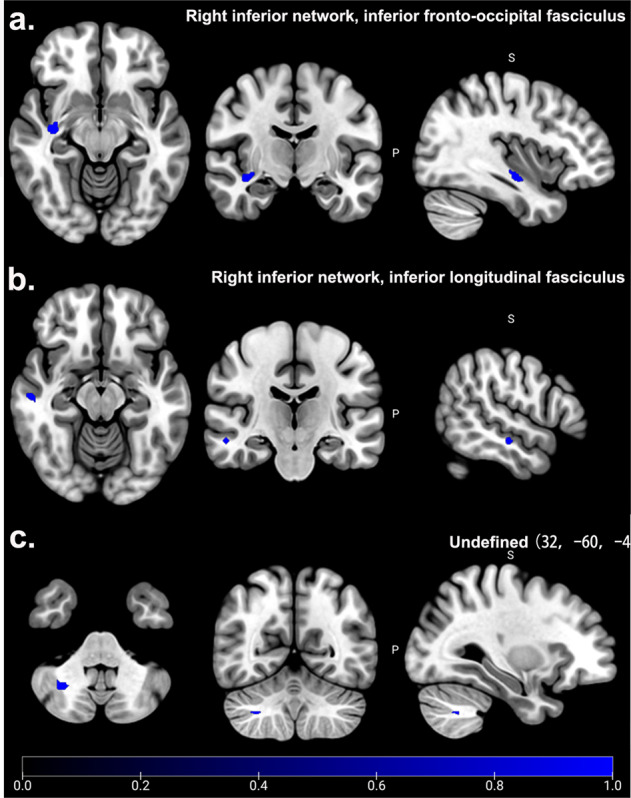
Significant WM abnormalities between T2DM-CD and HCs. Patients with T2DM-CD showed the significant and consistent decreased FA (Blue) in right inferior network, inferior fronto-occipital fasciculus (IFOF.R) (**a**), right inferior network, inferior longitudinal fasciculus (ILF.R) (**b**), and undefined area of cerebellum (32, −60, −42) (**c**) (*P* < 0.005). No increased FA was found in T2DM-CD compared to HCs (*P* = 0.005).

According to the main analysis, patients with T2DM-CD showed the significant and consistent decreased FA in right inferior network, inferior fronto-occipital fasciculus (IFOF.R) (*MNI*: 36, −12, −12, *SDM-Z* = −1.402, *P* < 0.001), right inferior network, longitudinal fasciculus (ILF.R) (*MNI*: 54, −18, −14, *SDM-Z* = −1.038, *P* < 0.005), and undefined area in cerebellum (*MNI*: 32, −60, −42, *SDM-Z* = −1.017, *P* < 0.005) compared to HCs (Table 2, Fig. 3). Q statistic showed no heterogeneity in main results (*Q* = 7.056, *P* > 0.05) (Supplementary Table 6, Supplementary Fig. 4). Jackknife analysis showed 4 of 5 times in right inferior network, inferior fronto-occipital fasciculus, 4 of 5 times in right inferior network, inferior longitudinal fasciculus, and 4 of 5 times in undefined area in cerebellum (Supplementary Table 7). Egger’s test was not performed to check the publication bias because of limited studies (<10 studies).

### Meta-regression analysis

**Fig. 4:**
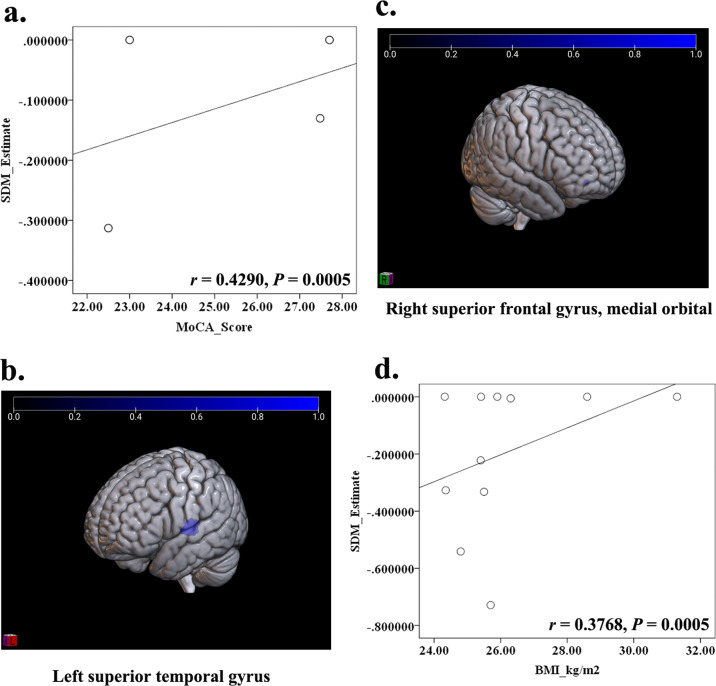
The relationship between MoCA score, BMI, and decreased GM in patients with T2DM-CD. According to the meta-regression, the MoCA score was positively related with the decreased GM in PFCventmed.R (**a**, **c**) (*r* = 0.4290, *P* < 0.0005), while the BMI was positively related with the decreased GM in STG.L (**b**, **d**) (*r* = 0.3768, *P* < 0.0005).

**Fig. 5:**
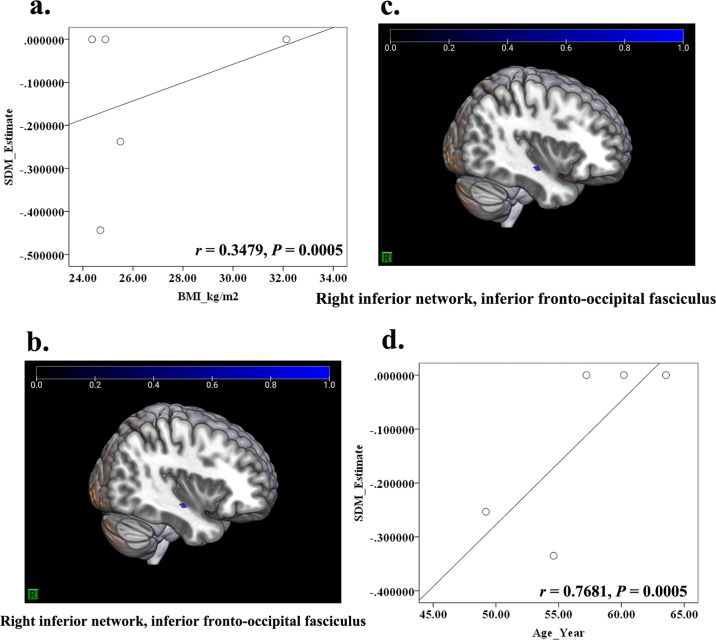
The relationship between BMI, age and decreased FA in patients with T2DM-CD. According to the meta-regression, both the BMI (**a**, **c**) (*r* = 0.3479, *P* < 0.0005) and age of patients (**b**, **d**) (*r* = 0.7681, *P* < 0.0005) were positively related with the decreased FA in IFOF.R.

The potential effects of risk factors (e.g., age, BMI, HbA1c, disease duration, and MMSE/MoCA score) on the GM or WM abnormalities in T2DM-CD were respectively performed in meta-regression. The results showed that the GM reduction in PFCventmed.R was positively related with the MoCA score, and the GM reduction in STG.L was positively related with the BMI (Table 3 and Fig. 4). In addition, the decreased FA in IFOF.R was positively related with both age and BMI (Table 3 and Fig. 5).

**Table 3:** The relationship between Age, BMI, MoCA and decreased GM/WM in patients with T2DM-CD.

Risk factors	MNI coordinate	*SDM-Z*	*P*	Voxels	Description
Gray matter					
BMI	−56,−18,10	−2.715	<0.0005	245	Left superior temporal gyrus, BA 48
MoCA	2,36, −14	−1.388	<0.0005	24	Right superior frontal gyrus, medial orbital, and BA 11
White matter					
BMI	36, −14, −10	−1.392	<0.0005	11	Right inferior network, inferior fronto-occipital fasciculus
Age	36, −12, −12	−1.827	<0.0005	18	Right inferior network, inferior fronto-occipital fasciculus

*BA* Brodmann area.

## Discussion

In our systemic review and meta-analysis, GM and WM abnormalities in patients with T2DM-CD were the first to be explored. The GMV, GMD, and cortical thickness changes between T2DM-CD and HCs were combined to explore the specific brain regions with GM alterations in T2DM-CD. The FA changes between T2DM-CD and HCs were pooled to explore the specific brain regions with WM abnormalities. Main analysis showed that patients with T2DM-CD had the significant decreased GM in PFCventmed.R (BA 11), STG.L (BA 48), and CAL.R (BA 17), as well, as they had decreased FA in IFOF.R, ILF.R, and undefined area of cerebellum area (32, −60, −42). Meta-regression showed that the BMI might be positively related with both decreased GM in STG.L and decreased FA in IFOF.R, the MoCA score might be positively related with the decreased GM in PFCventmed.R, and the age of patients might be positively related with the decreased FA in IFOF.R.

According to our main results, GM abnormalities in specific brain regions in patients with T2DM-CD were also consistent with the findings of previous systemic review and meta-analysis studies on brain structural alterations caused by T2DM. For example, previous meta-analysis of VBM studies reported that patients with T2DM had the significant GM reductions in bilateral STG, middle temporal gyrus (MTG), medial superior frontal gyrus (SFGmedial), insula (INS), median cingulate cortex, precuneus cortex, and the left lentiform nucleus [47]. Meanwhile, another meta-analysis study reported that the patients with T2DM showed a lower GMV in STG.L, MTG.R, right rolandic operculum (ROL.R), and left fusiform gyrus (FFG.L) than HCs [48]. In addition, the recent systemic review and meta-analysis also showed that the patients with T2DM had the significant decreased GMV in bilateral STG/ROL, MTG.L, ITG.L, and left supramarginal gyrus (SMG.L) [49]. Similarly, our study found that the patients with T2DM-CD had the significant decreased GM in STG.L compared to HCs. Taken together, the GM reduction in STG.L was robust in both T2DM and T2DM-CD. It was reported that the STG played a crucial role in extracting meaningful information from speech input [50]. Previous evidences also demonstrated that the functional deficit of STG was the common mechanism of CD caused by various diseases including T2DM, schizophrenia, and multiple sclerosis [37, 51, 52]. Therefore, the GM reduction in STG.L may be a specific biomarker for T2DM to T2DM-CD by impairing the function of speech information encoding.

Furthermore, our study also found that patients with T2DM-CD had the significant decreased FA in IFOF.R compared to HCs, which was consistent with the findings of previous systemic review and meta-analysis studies. For example, previous meta-analysis study reported that the patients with T2DM showed the significant decreased FA in IFOF.R, corpus callosum (CC), and left olfactory cortex compared to HCs [17]. Another same study found that patients with T2DM had the significant decreased FA in CC (genu and body), bilateral anterior and superior corona radiata (CR), bilateral cingulum, and bilateral superior fronto-occipital fasciculus (SFOF) [53]. Taken together, the decreased FA in FOF was robust in both T2DM and T2DM-CD. It was reported that the damaged WM integrity of IFOF.R was related with the episodic memory and attention function impairment in patients with T2DM [19]. And the previous studies have demonstrated that the FOF fiber tract was significantly involved in the linguistic, spatial, and visual functions [54–56]. Therefore, the decreased FA in FOF might be another specific biomarker for T2DM to T2DM-CD by impairing the memory and attention functions.

In addition to the robust results in both T2DM and T2DM-CD, our study also found that the patients with T2DM-CD had the significant GM reduction in PFCventmed.R (BA 11) and CAL.R (BA 17), as well as decreased FA in ILF.R and undefined area of cerebellum (32, −60, −42). Although previous studies have shown that the first three structural abnormalities were involved in the T2DM or CD [57–59], the further exploration in T2DM-CD was suggested. Furthermore, the decreased FA in undefined area of cerebellum (32, −60, −42) that was adjacent to the cerebellar dentate nucleus (CDN) was newly found in T2DM-CD. Previous studies reported that the abnormal functional connectivity between CDN and cerebral cortex was significantly correlated with the CD in Alzheimer’s disease (AD) or first-episode schizophrenia [60, 61]. Therefore, impaired information transmission resulting from decreased FA in the CDN-adjacent WM region might also be associated with T2DM-CD. Meta-regression showed that the risk factors of BMI, age of patients, and MoCA score might play an effect on the GM or WM alterations in T2DM-CD, but the results need to be verified due to the limited datasets. The other factors including MMSE score, percentage of female patients, HbA1c, and disease duration were suggested to be further explored in the future.

Some limitations in this study were declared as follows: Firstly, only published studies from 4 databases were searched; secondly, only peak coordinates and effect sizes of studies included were extracted and analyzed; thirdly, subgroup analysis was not performed because of limited datasets; fourthly, GMV, GMD, and cortical thickness were pooled into this meta-analysis, which might increase the heterogeneity; fifthly, the results of meta-regression should be accepted with caution because of the limited datasets.

In conclusion, GM and WM abnormalities caused by T2DM in specific brain regions (STG.L and FOF) were significantly correlated with the visual and linguistic dysfunctions, which might play a crucial role in the process of T2DM-CD. Our findings also showed that T2DM damages the GM and its functionally related WM (e.g., IFOF. R and PFCventmed.R), but not the adjacent WM. Those results might be a reference for researchers in the future studies.

## Supplementary information

Supplementary information

## Data Availability

All data included in this study is showed in this article or supplementary information, any further request is available by contacting with the corresponding authors.
