# Supplementary material for: Gray and white matter abnormality in patients with T2DM-related cognitive dysfunction: a systemic review and meta-analysis
Source: Nutr Diabetes. 2022 Aug 15;12:39. doi: 10.1038/s41387-022-00214-2 (PMC9378704; doi:10.1038/s41387-022-00214-2)
Supplement: Supplementary file 1 — Supplementary information [file 41387_2022_214_MOESM1_ESM.docx]

**Supplementary information**

**Contents**

DATA: The peak coordinates, effect sizes and their original studies………………...……………………………………………….….....1

Supplementary Table 1: Checklists of PRISMA statement for reporting systematic reviews and meta-analysis…………………………..8

Supplementary Table 2: The protocols of this systemic review and meta-analysis about GM and WM abnormality in PROSPERO……11

Supplementary Table 3: Detail search strategy of four databases………………………………………………………………………….27

Supplementary Table 4: Quality assessment of studies included by 12-point checklists……………………………………………..……30

Supplementary Table 5: The significant cognitive exanimation information of studies included……………………..…………………...32

Supplementary Table 6: Heterogeneity analysis of main results in GM/WM meta-analysis…………………………………………...….34

Supplementary Table 7: Jackknife analysis of main results in GM/WM meta-analysis…………………………………………………...38

Supplementary Fig. 1: The approach to pool the VBM and SBM studies into meta-analysis……………………………………………..41

Supplementary Fig. 2: Funnel plot of the robust main results in GM studies……………………………………………………………...42

Supplementary Fig. 3: Forest plot of the heterogeneity analysis in GM studies…………………………………………………………...43

Supplementary Fig. 4: Forest plot of the heterogeneity analysis in WM studies…………………………………………………………..44

Ethical statement…………………………………………………………………………………………………………………………...45

**DATA: The peak coordinates, effect size and their original studies**

| **Gray matter** | | | |
| --- | --- | --- | --- |
| **Number** | **Study** | **File name** | **Coordinate information (*x*, *y*, *z*, *t* value)** |
| 1 | Moran et al | Moran.no_peaks.txt | -14, 2, -16, -3.10  -64, -40, -10, -3.10  -64, -24, -15, -3.10  44, -22, 12, -3.10  -2, -35, 39, -3.10  -5, -54, 42, -3.10  8, 48, 2, -3.10  -7, 13, 9, -3.10  -20, 8, 5, -3.10  -40, -14, 11, -3.10  -49, -9, 6, -3.10  -54, 4, 20, -3.10  -1, 49, 2, -3.10  42, 42, 4, -3.10 |
| Moran C, Phan TG, Chen J, Blizzard L, Beare R, Venn A, Münch G, Wood AG, Forbes J, Greenaway TM, Pearson S, Srikanth V. Brain atrophy in type 2 diabetes: regional distribution and influence on cognition. Diabetes Care. 2013 Dec;36(12):4036-42. doi: 10.2337/dc13-0143. | | | |
| 2 | Li et al | Li.fsl_mni.txt | -43.2, 37.7, -14.0, -3.843  -43.9, -22.4, 21.1, -8.061  -39.2, -86.9, -7.5, -4.728  -10.1, -58.1, 11.4, -4.104  20.8, -63.1, 45.2, -3.588  53.8, -21.4, 19.5, -3.758  58.3, -34.3, -8.6, -4.241  41.0, -13.7, -12.0, -4.093  14.9, -53.9, 8.6, -4.493  5.3, -81.9, 21.9, -2.694 |
| Li C, Zuo Z, Liu D, Jiang R, Li Y, Li H, Yin X, Lai Y, Wang J, Xiong K. Type 2 Diabetes Mellitus May Exacerbate Gray Matter Atrophy in Patients With Early-Onset Mild Cognitive Impairment. Front Neurosci. 2020 Aug 11;14:856. doi: 10.3389/fnins.2020.00856. | | | |
| 3 | Chen et al | Chen.spm_mni.txt | -36, -61, 24, -18.46  35, 23, 39, -18.43  -20, 26, 39, -18.1  -41, -40, 57, -17.24  -41, 23, 21, -16.79  -62, -21, 36, -16.66  33, -43, 39, -13.07  39, 24, 19, -12.24  0, -88, 7, -12.12  32, 39, 13, -12.03  15, 47, -3, -12.03  -30, -72, 48, -11.23  8, 6, 32, -10.30 |
| Chen Z, Li J, Sun J, Ma L. Brain expansion in patients with type II diabetes following insulin therapy: a preliminary study with longitudinal voxel-based morphometry. J Neuroimaging. 2014 Sep-Oct;24(5):484-91. doi: 10.1111/jon.12059. | | | |
| 4 | Natalia et al | Natalia.spm_tal.txt | -20, 7, 64, -6.15  -4, 24, -18, -5.13  4, 28, -13, -4.73  16, 15, -14, -4.66  -16, 2, -29, -4.61  14, -4, -35, -4.72  26, 29, 2, -4.09  -12, -86, -41, -4.08  -14, 15, -16, -6.56  22, 29, 32, -4.31  16, 62, -1, -5.31  -12, 18, -19, -4.91  -6, 41, 33, -4.72  53, -5, -18, -4.75  32, -7, 21, -4.11  -22, 11, 57, -5.81  -34, 2, -39, -4.48  59, -19, 43, -4.26  -32, 35, -3, -4.72  34, -55, 18, -4.50  -8, -26, 31, -4.33  -24, -50, -23, -4.33  -14, -83, -21, -5.13  4, -59, -21, -4.17  22, 29, 34, -4.87  -16, -73, 46, -4.72  46, -18, -11, -4.24  -14, -59, -46, -3.37 |
| García-Casares N, Jorge RE, García-Arnés JA, Acion L, Berthier ML, Gonzalez-Alegre P, Nabrozidis A, Gutiérrez A, Ariza MJ, Rioja J, González-Santos P. Cognitive dysfunctions in middle-aged type 2 diabetic patients and neuroimaging correlations: a cross-sectional study. J Alzheimers Dis. 2014;42(4):1337-46. doi: 10.3233/JAD-140702. | | | |
| 5 | Wang et al | Wang.other_mni.txt | 62, -39, 12, -5.54  62, -48, 27, -5.04  -35, -79, -6, -4.25  56, -42, -29, -3.82 |
| Wang C, Fu K, Liu H, Xing F, Zhang S. Brain structural changes and their correlation with vascular disease in type 2 diabetes mellitus patients: a voxel-based morphometric study. Neural Regen Res. 2014 Aug 15;9(16):1548-56. doi: 10.4103/1673-5374.139482. | | | |
| 6 | Yoon et al | Yoon.fsl_tal.txt | 42.1, -59.7, -5.7, -3.18  51.2, -35.6, 8.3, -3.18  14.3, -60.5, 5.1, -3.18  10.3, 3.9, 45.6, -2.08  -40.8, -50.2, -10.0, -2.56  -55.7, -48.3, -28.4, -3.18  -7.5, 26.0, 35.4, -2.71  -10.8, -62.9, 5.1, -2.71  -54.8, -7.1, 9.6, -3.18 |
| Yoon S, Cho H, Kim J, Lee DW, Kim GH, Hong YS, Moon S, Park S, Lee S, Lee S, Bae S, Simonson DC, Lyoo IK. Brain changes in overweight/obese and normal-weight adults with type 2 diabetes mellitus. Diabetologia. 2017 60(7):1207-1217. doi: 10.1007/s00125-017-4266-7. | | | |
| 7 | Zhang et al | Zhang.spm_mni.txt | -57, -22, -5, -4.1  66, -21, -5, -4.00  41, 11, -39, -4.64  -39, 6, -32, -4.40  -33, -69, -11, -5.56  41, -55, -21, -4.06  -35, -84, 0, -4.46  32, -82, 17, -4.9  23, -93, -17, -3.62  -41, -86, -6, -3.53  -14, -82, 42, -3.68  18, -49, 52, -4.76  41, -52, 57, -3.94  18, 57, -12, -4.55  -9, -30, 33, -4.76  -29, 9, -23, -3.89  -30, -1, -29, -3.31  -35, -16, -27, -3.24  17, -32, -3, -4.16 |
| Zhang Y, Zhang X, Zhang J, Liu C, Yuan Q, Yin X, Wei L, Cui J, Tao R, Wei P, Wang J. Gray matter volume abnormalities in type 2 diabetes mellitus with and without mild cognitive impairment. Neurosci Lett. 2014 562:1-6. doi: 10.1016/j.neulet.2014.01.006. | | | |
| 8 | Zhou et al | Zhou.spm_tal.txt | -33, 32, 34, -6.06  30, 35, 33, -5.41  43, 13, -3, -4.00  -53, -38, 11, -4.69  -57, -19, -7, -4.63  41, 13, -17, -4.20  38, -27, 13, -3.89  -43, -12, 13, -5.49  -56, -31, 30, -4.78  20, -32, 55, -4.30  -56, -13, 29, -4.44  -4, 29, -3, -4.86  -17, -66, 14, -4.58  17, -60, 3, -5.68  20, -75, 22, -4.43  -22, -47, -13, -4.75  22, -25, -19, -4.82 |
| H. Zhou, W. Lu, Z. Zhang, F. Bai, J. Chang, and G. Teng, Study of cognitive function and brain volume in type 2 diabetic patients. Zhonghua yi xue za zhi 90 (2010) 327-331. | | | |
| 9 | Wei et al | Wei.spm_mni.txt | 27, -65, -54, -3.57  -56, -20, -21, -4.01  -24, -102, 6, -3.40 |
| T. Wei, X. Liu, D. Cui, Y. Xue, Y. Guo, A. Lu, W. Cao, Y. Guo, and Q. Jiao, Gray matter density alteration and its correlation with cognitive impairment in patients with type 2 diabetes mellitus. Chinese Journal of Medical Physics 35 (2018) 364-368. | | | |
| 10 | Duan et al | Duan.spm_mni.txt | 3, 36, -15, -1.66 |
| S. Duan, D. Liu, T. Li, J. Kuang, P. Wei, and J. Wang, Effect of type 2 diabetes mellitus on cognitive function in middle-aged adults and its characteristics. Journal of Third Military Medical University 38 (2016) 314-319. | | | |
| 11 | Feng et al | Feng.spm_mni.txt | 12, 70.5, 3, -5.1108  -39, 42, -9, -5.0316 |
| Feng Y, Li Y, Tan X, Liang Y, Ma X, Chen Y, Lv W, Wu J, Kang S, Li M, Qiu S. Altered Gray Matter Volume, Functional Connectivity, and Degree Centrality in Early-Onset Type 2 Diabetes Mellitus. Front Neurol. 2021 12:697349. doi: 10.3389/fneur.2021.697349. | | | |
| **White matter** | | | |
| **Number** | **Study** | **File name** | **Coordinate information (*x*, *y*, *z*, *t* value)** |
| 1 | Cui et al | Cui.fsl_mni.txt | 6, -87, 3, -3.85  -6, -87, 3, -3.85  15, -24, 81, -3.27  -15, -24, 81, -3.27 |
| Cui Y, Tang TY, Lu CQ, Lu T, Wang YC, Teng GJ, Ju S. Disturbed Interhemispheric Functional and Structural Connectivity in Type 2 Diabetes. J Magn Reson Imaging. 2022 Feb;55(2):424-434. doi: 10.1002/jmri.27813. | | | |
| 2 | Kim et al | Kim.fsl_mni.txt | 34, -60, 1, -3.374  33, -48, 15, -3.085  -28, -63, 17, -3.946  36, -28, 3, -2.799  39, -38, -3, -2.803  26, -53, 16, -3.071  17, -35, 33, -2.636  28, -27, -4, -3.568  43, -29, -12, -3.279  35, -17, -7, -2.059 |
| Kim DJ, Yu JH, Shin MS, Shin YW, Kim MS. Hyperglycemia Reduces Efficiency of Brain Networks in Subjects with Type 2 Diabetes. PLoS One. 2016 11(6): e0157268. doi: 10.1371/journal.pone.0157268. | | | |
| 3 | Xiong et al | Xiong.no_peaks.txt | 47, 133, 50, -2.41 |
| Xiong Y, Zhang S, Shi J, Fan Y, Zhang Q, Zhu W. Application of neurite orientation dispersion and density imaging to characterize brain microstructural abnormalities in type-2 diabetics with mild cognitive impairment. J Magn Reson Imaging. 2019 50(3):889-898. doi: 10.1002/jmri.26687. | | | |
| 4 | Yau et al | Yau.other_tal.txt | 45, 34, -32, -3.33  -38, -21, -14, -3.54  37, 43, -13, -3.27  -30, 3, -16, -3.86  49, 58, 12, -3.69  54, 53, -28, -3.34 |
| Yau PL, Javier D, Tsui W, Sweat V, Bruehl H, Borod JC, Convit A. Emotional and neutral declarative memory impairments and associated white matter microstructural abnormalities in adults with type 2 diabetes. Psychiatry Res. 2009 174(3):223-30. doi: 10.1016/j.pscychresns.2009.04.016. | | | |
| 5 | Yoon et al | Yoon.other_mni.txt | -39, -8, -18, -1.66  -5, 27, 1, -1.66  13, -22, -17, -1.66  -6, -9, 10, -1.66  18, 12, 35, -1.66  -23, -70, -32, -1.66  22, -71, -32, -1.66  21, 44, 17, -1.66  24, -78, 23, -1.66  29, 13, 7, -1.66  36, -11, -12, -1.66  -20, 35, 8, -1.66  21, -34, 51, -1.66  -34, -55, -44, -1.66  31, -60, -42, -1.66  22, -86, 4, -1.66  19, -39, 30, -1.66  -24, -75, 13, -1.66  37, -76, 1, -1.66  -30, 10, 1, -1.66  23, -46, 48, -1.66  57, -17, -15, -1.66 |
| Yoon S, Cho H, Kim J, Lee DW, Kim GH, Hong YS, Moon S, Park S, Lee S, Lee S, Bae S, Simonson DC, Lyoo IK. Brain changes in overweight/obese and normal-weight adults with type 2 diabetes mellitus. Diabetologia. 2017 60(7):1207-1217. doi: 10.1007/s00125-017-4266-7. | | | |

| **Table 1. Checklists of PRISMA statement for reporting systematic reviews and meta-analysis** | | | |  |
| --- | --- | --- | --- | --- |
| **Section/topic** | **No.** | **Checklists** | **Reported on page No.** |  |
| **Title** | | | | |
| Title | 1 | Identify the report as a systematic review, meta-analysis, or both | 1 |  |
| **Abstract** | | | | |
| Structured summary | 2 | Provide a structured summary including, as applicable, background, objectives, data  sources, study eligibility criteria, participants, interventions, study appraisal and synthesis  methods, results, limitations, conclusions and implications of key findings, systematic  review registration number | 2 |  |
| **Introduction** | | | |  |
| Rationale | 3 | Describe the rationale for the review in the context of what is already known | 1-2 |  |
| Objectives | 4 | Provide an explicit statement of questions being addressed with reference to participants,  interventions, comparisons, outcomes, and study design (PICOS) | 2 |  |
| **Methods** | | | |  |
| Protocol and registration | 5 | Indicate if a review protocol exists, if and where it can be accessed (such as web address),  and, if available, provide registration information including registration number | 2 |  |
| Eligibility criteria | 6 | Specify study characteristics (such as PICOS, length of follow-up) and report characteristics (such as years considered, language, publication status) used as criteria for eligibility, giving rationale | 2 |  |
| Information sources | 7 | Describe all information sources (such as databases with dates of coverage, contact with  study authors to identify additional studies) in the search and date last searched | 2 |  |
| Search | 8 | Present full electronic search strategy for at least one database, including any limits used,  such that it could be repeated | 2 |  |
| Study selection | 9 | State the process for selecting studies (that is, screening, eligibility, included in systematic  review, and, if applicable, included in the meta-analysis) | 2 |  |
| Data collection process | 10 | Describe method of data extraction from reports (such as piloted forms, independently, in  duplicate) and any processes for obtaining and confirming data from investigators | 2 |  |
| Data items | 11 | List and define all variables for which data were sought (such as PICOS, funding sources) and any assumptions and simplifications made | 2 |  |
| Risk of bias in individual studies | 12 | Describe methods used for assessing risk of bias of individual studies (including  specification of whether this was done at the study or outcome level), and how this  information is to be used in any data synthesis | 2 |  |
| Summary measures | 13 | State the principal summary measures (such as risk ratio, difference in means). | 2 |  |
| Synthesis of results | 14 | Describe the methods of handling data and combining results of studies, if done, including  measures of consistency (such as I2) for each meta-analysis | 2 |  |
| Risk of bias across studies | 15 | Specify any assessment of risk of bias that may affect the cumulative evidence (such as  publication bias, selective reporting within studies) | 2 |  |
| Additional analyses | 16 | Describe methods of additional analyses (such as sensitivity or subgroup analyses, meta-regression), if done, indicating which were pre-specified | 2 |  |
| **Results** | | | |  |
| Study selection | 17 | Give numbers of studies screened, assessed for eligibility, and included in the review, with reasons for exclusions at each stage, ideally with a flow diagram | 4 |  |
| Study characteristics | 18 | For each study, present characteristics for which data were extracted (such as study size,  PICOS, follow-up period) and provide the citations | 4 |  |
| Risk of bias within studies | 19 | Present data on risk of bias of each study and, if available, any outcome-level assessment  (see item 12). | 4 |  |
| Results of individual studies | 20 | For all outcomes considered (benefits or harms), present for each study (a) simple summary data for each intervention group and (b) effect estimates and confidence intervals, ideally with a forest plot | 4 |  |
| Synthesis of results | 21 | Present results of each meta-analysis done, including confidence intervals and measures of consistency | 4 |  |
| Risk of bias across studies | 22 | Present results of any assessment of risk of bias across studies (see item 15) | 4 |  |
| Additional analysis | 23 | Give results of additional analyses, if done (such as sensitivity or subgroup analyses, meta-regression [see item 16]) | 4-5 |  |
| Summary of evidence | 24 | Summarise the main findings including the strength of evidence for each main outcome;  consider their relevance to key groups (such as health care providers, users, and policy  makers) | 5-7 |  |
| Limitations | 25 | Discuss limitations at study and outcome level (such as risk of bias), and at review level (such as incomplete retrieval of identified research, reporting bias) | 7 |  |
| Conclusions | 26 | Provide a general interpretation of the results in the context of other evidence, and  implications for future research | 7 |  |
| **Funding** | | | |  |
| Funding | 27 | Describe sources of funding for the systematic review and other support (such as supply of data) and role of funders for the systematic review | 7 |  |

| **Table 2. The protocols of this systemic review and meta-analysis about GM and WM abnormality in PROSPERO** |
| --- |
| **1) GM systemic review and meta-analysis protocol in PROSPERO** |
| **1. * Review title.**  Cerebral cortex abnormality in type2 diabetes mellitus patients with cognitive dysfunction: A systemic review and meta-analysis  **3. * Anticipated or actual start date.**  Give the date the systematic review started or is expected to start.  15/04/2021  **4. * Anticipated completion date.**  Give the date by which the review is expected to be completed.  31/12/2021  **5. * Stage of review at time of this submission.**  The review has not yet started: No   \| **Review stage** \| **Started** \| **Completed** \| \| --- \| --- \| --- \| \| Preliminary searches \| Yes \| No \| \| Piloting of the study selection process \| Yes \| No \| \| Formal screening of search results against eligibility criteria \| No \| No \| \| Data extraction \| No \| No \| \| Risk of bias (quality) assessment \| No \| No \| \| Data analysis \| No \| No \|   **6. * Named contact.**  Teng Ma  **7. * Named contact email.**  875728314@qq.com  **10. * Organisational affiliation of the review.**  Department of Radiology & Functional and Molecular Imaging Key Lab of Shaanxi Province, Tangdu Hospital, Fourth Military Medical University (Air Force Medical University)  **11. * Review team members and their organisational affiliations.**  Mr Teng Ma. Department of Radiology & Functional and Molecular Imaging Key Lab of Shaanxi Province, Tangdu Hospital, Fourth Military Medical University (Air Force Medical University)  Mr Zeyang Li. Department of Radiology & Functional and Molecular Imaging Key Lab of Shaanxi Province, Tangdu Hospital, Fourth Military Medical University (Air Force Medical University)  **12. * Funding sources/sponsors.**  Department of Radiology & Functional and Molecular Imaging Key Lab of Shaanxi Province, Tangdu Hospital, Fourth Military Medical University (Air Force Medical University)  **13. * Conflicts of interest.**  None  **15. * Review question.**  The relationship between brain structure abnormality with cognitive impairment in type 2 diabetes mellitus (T2DM) has been searched in quite a few studies. The changed cerebral cortex has been reported by researchers in variable regions of brain, but the most significant area can be still unclear. This article aims to review the previous studies about the cerebral cortex alternation in T2DM with cognitive dysfunction and make a meta-analysis to find the most significant region, which can be a reference for more researcher to understand neuroimaging mechanism of cognitive impairment within T2DM.  **16. * Searches.**  PubMed, MEDLINE, Web of Science and Cochrane Library will be searched, and the search deadline is April 15, 2021. Besides, the references will be a supplement way to search relative studies. The participants are adults (include male and female).  **18. * Condition or domain being studied.**  Type 2 diabetes mellitus (T2DM) has been a worldwide health challenge and the number of populations is growing yearly. Study has shown that T2DM can cause a series of complications, such as cerebrocardiovascular disease, renal disease, diabetic retinopathy and so on. T2DM is also an important risk for cognitive dysfunction and a large number of studies confirmed the change of brain structure. It is reported that T2DM could contribute to cognitive impairment by affecting the brain volume. It is also certified that the changed cortical thickness of T2DM could affect the cognitive function. Previous systemic review and meta-analysis had explored the abnormality gray matter between T2DM with healthy controls (HCs), while there has no meta-analysis focused on the changed cerebral cortex in T2DM with cognitive dysfunction. This study aims to collect the studies about the abnormality cerebral cortex in T2DM with cognitive dysfunction compared with HCs and make a meta-analysis to find the significant abnormal brain region and give a reference for researchers in learning the neuroimaging mechanism of cognitive impairment caused by T2DM in the future.  **19. * Participants/population.**  T2DM patients with cognitive impairment  **20. * Intervention(s), exposure(s).**  The white matter impaired in cognitive dysfunction patients caused by T2DM.  **21. * Comparator(s)/control.**  Healthy controls  **22. * Types of study to be included.**  Studies compared the T2DM with cognitive dysfunction patients’ white matter (Diffusion tension imaging (DTI)) abnormality with healthy control, and the positive coordination was reported in standard space (such as MNI or Tal space) will be included.  **24. * Main outcome(s).**  Find the most significant coordination of whiter matter in standard space (such as MNI or TAL) after meta-analysis by AES-SDM software (include mean analysis and Jackknife analysis).  **25. * Additional outcome(s).**  The additional outcomes include subgroup analysis and meta-regression, whether make the additional analysis will be dependent on the factual situation.  **26. * Data extraction (selection and coding).**  Studies from PubMed, MEDLINE, Cochrane Library and Web of Science are searched and evaluated by two authors independently, any disagreement will be resolved by consulting a third author, and the results are documented in EndNote software to select and exclude the studies.  Including criteria:  (1) Reported the whiter matter abnormality in T2DM with cognitive dysfunction.  (2) Comparison between T2DM patients with HCs.  (3) Adults include male and female, and age>18years old.  (4) Show the available coordinates information in standard space (such as Montreal Neurological Institute (MNI) and Talairach space) and the effect size (such as Z score or T value). The coordinate information of included studies (such as MNI or TAL x, y, z), and effect size (such as T value or Z score) in the article included. All of coordinate information will be recorded in specific format of AES-SDM software requirement. Besides, general information, including the first author, sample size (male and female), age (mean ± sd.), MRI scan parameters and so on will be recorded and reported in this article.  **27. * Risk of bias (quality) assessment.**  The risk of bias will be assessed by the AES-SDM software version 5.15 (https://www.sdmproject.com). We will create a mask according to the significant abnormal white matter fibers. Then extract the information and make a funnel plot.  **28. * Strategy for data synthesis.**  The data extracted from studies (include peak coordinates information and T value) will be recorded respectively with specific format required by AES-SDM software. The detail data analysis steps are as follows:  First, Global analysis  Second, Pre-processing  Third, Mean analysis  Fourth, Threshold  Fifth, Jackknife analysis will be implemented after the main analysis being finished  Sixth, Extract information  Seventh, Bias Test and make a funnel plot  Eighth, meta-regression (according to the factual situation)  **29. * Analysis of subgroups or subsets.**  Meta-regression and subgroup analysis will be performed according to the factual situation  **30. * Type and method of review.**   \| Meta-analysis \| Yes \| \| --- \| --- \| \| Systematic review \| Yes \|   **32. * Country.**  China  **38. * Current review status.**  Review_Ongoing |

| **2) WM systemic review and meta-analysis protocol in PROSPERO** |
| --- |
| **1. * Review title.**  White matter abnormality in type 2 diabetes mellitus patients with cognitive dysfunction: A systemic review and meta-analysis  **3. * Anticipated or actual start date.**  15/05/2021  **4. * Anticipated completion date.**  31/03/2022  **5. * Stage of review at time of this submission.**  The review has not yet started: No   \| **Review stage** \| **Started** \| **Completed** \| \| --- \| --- \| --- \| \| Preliminary searches \| Yes \| No \| \| Piloting of the study selection process \| Yes \| No \| \| Formal screening of search results against eligibility criteria \| No \| No \| \| Data extraction \| No \| No \| \| Risk of bias (quality) assessment \| No \| No \| \| Data analysis \| No \| No \|   **6. * Named contact.**  Teng Ma  **7. * Named contact email.**  875728314@qq.com  **10. * Organisational affiliation of the review.**  Department of Radiology & Functional and Molecular Imaging Key Lab of Shaanxi Province, Tangdu Hospital, Fourth Military Medical University (Air Force Medical University)  **11. * Review team members and their organisational affiliations.**  Mr Teng Ma. Department of Radiology & Functional and Molecular Imaging Key Lab of Shaanxi Province, Tangdu Hospital, Fourth Military Medical University (Air Force Medical University)  Mr Zeyang Li. Department of Radiology & Functional and Molecular Imaging Key Lab of Shaanxi Province, Tangdu Hospital, Fourth Military Medical University (Air Force Medical University)  **12. * Funding sources/sponsors.**  Department of Radiology & Functional and Molecular Imaging Key Lab of Shaanxi Province, Tangdu Hospital, Fourth Military Medical University (Air Force Medical University)  **13. * Conflicts of interest.**  None  **15. * Review question.**  White matter impaired in type 2 diabetes mellitus (T2DM) with and without cognitive dysfunction had been reported in past decades. A series of fibers reported in researches are showed abnormality and there is lack of a consistent significant result for T2DM with cognitive dysfunction. In this article, we are going to collect the previous studies and make a meta-analysis to find the most significant fiber changed in T2DM with cognitive dysfunction. So, this work could give a reference for researchers to understand the brain structure changed in this disease with cognition impaired complication.  **16. * Searches.**  PubMed, MEDLINE, Web of Science and Cochrane Library will be searched, and the search deadline is May 15, 2021. Besides, the references will be a supplement way to search relative studies. The participants are adults include male and female.  **18. * Condition or domain being studied.**  Since the population of T2DM has been enlarged year by year, people with T2DM are suffering the effect of brain structure abnormality. In the previous studies, T2DM could affect the white matter fiber and contribute to the cognitive decline. Because of various results reported and lacking of consistent significant white matter fiber, a systemic review and meta-analysis is necessary to find the most significant abnormality of T2DM with cognitive dysfunction. The results of this study will give more researchers a reference to understand the mechanism of  brain structure changed within T2DM with cognitive dysfunction.  **19. * Participants/population.**  T2DM patients with cognitive impairment  Including criteria:  (1) Reported the whole-brain cortex abnormality in T2DM.  (2) Comparison between T2DM patients with HCs.  (3) Adults include male and female and age >18 years old.  (4) Show the available coordinates information in standard space, such as Montreal Neurological Institute (MNI) and Talairach space.  (5) All individuals made psychological examinations and the T2DM show cognitive dysfunction and HCs without cognitive impairment.  Excluding criteria:  (1) Lack HCs, single sex, adolescences, T2DM without cognitive dysfunction and regions-of-interest (ROIs) analysis.  (2) Animal experiment, unrelated diseases, review, case report, letters, and another unrelated article  (3) White matter, function magnetic resonance imaging (fMRI), and any other do not match the including criteria.  **20. * Intervention(s), exposure(s).**  The cerebral cortex abnormality in cognitive dysfunction patients caused by T2DM.  Including criteria:  (1) Reported the whole-brain cortex abnormality in T2DM.  (2) Comparison between T2DM patients with HCs.  (3) Adults include male and female and age >18 years old.  (4) Show the available coordinates information in standard space, such as Montreal Neurological Institute (MNI) and Talairach space.  (5) All individuals receipted psychological examinations and the T2DM show cognitive dysfunction and HCs without cognitive impairment.  Excluding criteria:  (1) Lack HCs, single sex, adolescences, T2DM without cognitive dysfunction and regions-of-interest (ROIs) analysis.  (2) Animal experiment, unrelated diseases, review, case report, letters, and another unrelated article  (3) White matter, function magnetic resonance imaging (fMRI), and any other don’t match the including criteria.  **21. * Comparator(s)/control.**  Healthy controls  Including criteria:  (1) Reported the whole-brain cortex abnormality in T2DM.  (2) Comparison between T2DM patients with HCs.  (3) Adults include male and female and age >18 years old.  (4) Show the available coordinates information in standard space, such as Montreal Neurological Institute (MNI) and Talairach space.  (5) All individuals receipted psychological examinations and the T2DM show cognitive dysfunction and HCs without cognitive impairment.  Excluding criteria:  (1) Lack HCs, single sex, adolescences, T2DM without cognitive dysfunction and regions-of-interest (ROIs) analysis.  (2) Animal experiment, unrelated diseases, review, case report, letters, and another unrelated article  (3) White matter, function magnetic resonance imaging (fMRI), and any other don’t match the including criteria.  **22. * Types of study to be included.**  Studies compared the T2DM with cognitive dysfunction patients ’global cerebral cortex (such as the gray matter atrophy, cortical thickness changed and gray matter density abnormal) with healthy control, and the positive coordination was reported in standard space (such as MNI or Tal space) will be included.  **24. * Main outcome(s).**  Find the most significant coordination in standard space (such as MNI or TAL) after meta-analysis by AES-SDM software (include global analysis, preprocessing, mean analysis and Jackknife analysis, threshold, funnel plot).  **25. * Additional outcome(s).**  The additional outcomes include subgroup analysis and meta-regression, whether make the additional analysis will be dependent on the factual situation.  **26. * Data extraction (selection and coding).**  Studies from PubMed, MEDLINE, Cochrane Library and Web of Science are searched and evaluated by two authors independently, any disagreement will be resolved by consulting a third author, and the results are documented in EndNote software to select and exclude the studies.  Including criteria:  (1) Reported the whole-brain cortex abnormality in T2DM.  (2) Comparison between T2DM patients with HCs.  (3) Adults include male and female and age >18 years old.  (4) Show the available coordinates information in standard space, such as Montreal Neurological Institute (MNI) and Talairach space.  (5) All individuals receipted psychological examinations and the T2DM show cognitive dysfunction and HCs without cognitive impairment. The coordinate information of included studies (such as MNI or TAL x, y, z), and effect size (such as T value or Z score) in the article included. All of coordinate information will be recorded in specific format of AES-SDM  software requirement. Besides, general information, including the first author, sample size (male and female), age (mean ± sd.), MRI scan parameters and so on will be recorded and reported in this article.  **27. * Risk of bias (quality) assessment.**  The risk of bias will be assessed by the AES-SDM software version 5.15 (https://www.sdmproject.com). Extracting values from relevant peaks and check their funnel plots.  **28. * Strategy for data synthesis.**  The data of each study (include peak coordinates information and T value) will be recorded respectively with specific format required by AES-SDM software. The detail data analysis steps are as follows:  First, Global analysis  Second, Pre-processing  Third, Mean analysis + Jackknife analysis  Fourth, Threshold analysis  Fifth, Extract  Sixth, Bias Test  **29. * Analysis of subgroups or subsets.**  Meta-regression and subgroup analysis will be performed according to the factual situation  **30. * Type and method of review.**   \| Meta-analysis \| Yes \| \| --- \| --- \| \| Systematic review \| Yes \|   **32. * Country.**  China  **38. * Current review status.**  Review_Ongoing |

| **Table 3. Detailed search strategy of four databases** | |
| --- | --- |
| **Gray matter** | |
| **PubMed** | ((((((Diabetes Mellitus, Type 2[MeSH Terms]) OR (Diabetes Mellitus, Type 2[Text Word]) OR (Type 2 Diabetes[Text Word])) OR (NIDDM[Text Word])) OR (T2DM[Text Word])) OR (Diabetes Mellitus, Type II[Text Word])) AND (((((Cognitive Dysfunction[MeSH Terms]) OR (Cognitive Dysfunction[Text Word])) OR (cognitive decline[Text Word])) OR (cognitive impairment[Text Word])) OR (cognitive dissonance[Text Word]))) AND ((((((((((Cerebral Cortex[MeSH Terms]) OR (Cerebral Cortex[Text Word])) OR (Gray Matter[MeSH Terms])) OR (Gray Matter[Text Word]) ) OR (VBM[Text Word])) OR (voxel-based morphometry[Text Word])) OR (cortical thickness[Text Word])) OR (thickness[Text Word])) OR (cortical thinning[Text Word])) OR (thinning[Text Word])) |
| **Web of Science** | (TS=Diabetes Mellitus, Type 2 OR TS=Type 2 Diabetes OR TS=NIDDM OR TS=T2DM OR TS=Diabetes Mellitus, Type II) AND (TS=Cognitive Dysfunction OR TS=cognitive decline OR TS=cognitive impairment OR TS=cognitive dissonance) AND (TS=Cerebral Cortex OR TS=Gray Matter OR TS=VBM OR TS=voxel-based morphometry OR TS=cortical thickness OR TS=thickness OR TS=cortical thinning OR TS=thinning) |
| **MEDLINE** | ((Diabetes Mellitus, Type 2 or type 2 diabetes or NIDDM or T2DM or Diabetes Mellitus, Type II).tw.) AND ((Cognitive Dysfunction or cognitive decline or cognitive impairment or cognitive dissonance).tw.) AND ((cerebral cortex or gray matter or VBM or voxel-based morphometry or cortical thickness or thickness or cortical thinning or thinning).tw.) |
| **Cochrane library** | # 1 MeSH descriptor: [Diabetes Mellitus, Type 2] explode all trees  # 2 (Diabetes Mellitus, Type 2): ti,ab,kw  # 3 (Type 2 Diabetes): ti,ab,kw  # 4 (NIDDM): ti,ab,kw  # 5 (T2DM): ti,ab,kw  # 6 (Diabetes Mellitus, Type II): ti,ab,kw  # 7 MeSH descriptor: [Cognitive Dysfunction] explode all trees  # 8 (Cognitive Dysfunction): ti,ab,kw  # 9 (cognitive decline): ti,ab,kw  # 10 (cognitive impairment): ti,ab,kw  # 11 (cognitive dissonance): ti,ab,kw  # 12 MeSH descriptor: [Cerebral Cortex] explode all trees  # 13 (Cerebral Cortex): ti,ab,kw  # 14 MeSH descriptor: [Gray Matter] explode all trees  # 15 (Gray Matter): ti,ab,kw  # 16 (VBM): ti,ab,kw  # 17 (voxel-based morphometry): ti,ab,kw  # 18 (cortical thickness): ti,ab,kw  # 19 (cortical thinning): ti,ab,kw  # 20 (thinning): ti,ab,kw  (#1 or #2 or #3 or #4 or #5 or #6) and (#7 or #8 or #9 or #10 or #11) and (#12 or #13 or #14 or #15 or #16 or #17 or #18 or #19 or #20) |
| **White matter** | |
| **PubMed** | ((Diabetes Mellitus, Type 2[MeSH Terms] OR Diabetes Mellitus, Type 2[Text Word] OR (Type 2 Diabetes[Text Word] OR NIDDM[Text Word] OR (T2DM[Text Word])) OR Diabetes Mellitus, Type II[Text Word]) AND (Cognitive Dysfunction[MeSH Terms] OR Cognitive Dysfunction[Text Word] OR cognitive decline[Text Word] OR cognitive impairment[Text Word] OR cognitive dissonance[Text Word])) AND (white matter[MeSH Terms] OR white matter[Text Word] OR fractional anisotropy[Text Word] OR FA[Text Word] OR mean diffusivity[Text Word] OR MD[Text Word] OR VBA[Text Word] OR TBSS[Text Word] OR voxel-based analysis[Text Word] OR Tract-Based Spatial Statistics[Text Word]) |
| **Web of Science** | (TS=Diabetes Mellitus, Type 2 OR TS=Type 2 Diabetes OR TS=NIDDM OR TS=T2DM OR TS=Diabetes Mellitus, Type II) AND (TS=Cognitive Dysfunction OR TS=cognitive decline OR TS=cognitive impairment OR TS=cognitive dissonance) AND (TS=white matter OR TS=fractional anisotropy OR TS=FA OR TS=mean diffusivity OR TS=MD OR TS=VBA OR TS=TBSS OR TS=voxel-based analysis OR TS=Tract-Based Spatial Statistics) |
| **MEDLINE** | (Diabetes Mellitus, Type 2 or type 2 diabetes or NIDDM or T2DM or Diabetes Mellitus, Type II).tw. AND (Cognitive Dysfunction or cognitive decline or cognitive impairment or cognitive dissonance).tw. AND (White matter or fractional anisotropy or FA or mean diffusivity or MD or VBA or TBSS or voxel-based analysis or Tract-Based Spatial Statistics).tw. |
| **Cochrane library** | # 1 MeSH descriptor: [Diabetes Mellitus, Type 2] explode all trees  # 2 (Diabetes Mellitus, Type 2): ti,ab,kw  # 3 (Type 2 Diabetes): ti,ab,kw  # 4 (NIDDM): ti,ab,kw  # 5 (T2DM): ti,ab,kw  # 6 (Diabetes Mellitus, Type II): ti,ab,kw  # 7 MeSH descriptor: [Cognitive Dysfunction] explode all trees  # 8 (Cognitive Dysfunction): ti,ab,kw  # 9 (cognitive decline): ti,ab,kw  # 10 (cognitive impairment): ti,ab,kw  # 11 (cognitive dissonance): ti,ab,kw  # 12 MeSH descriptor: [White Matter] explode all trees  # 13 (white matter): ti,ab,kw  # 14 (fractional anisotropy): ti,ab,kw  # 15 (FA): ti,ab,kw  # 16 (mean diffusivity): ti,ab,kw  # 17 (MD): ti,ab,kw  # 18 (VBA): ti,ab,kw  # 19 (TBSS): ti,ab,kw  # 20 (voxel-based analysis): ti,ab,kw  # 21 (Tract-Based Spatial Statistics): ti,ab,kw  (#1 or #2 or #3 or #4 or #5 or #6) and (#7 or #8 or #9 or #10 or #11) and (#12 or #13 or #14 or #15 or #16 or #17 or #18 or #19 or #20 or #21) |

| **Table 4. Quality assessment of 15 studies included by 12-point checklists** | | | | | | | | | | | |
| --- | --- | --- | --- | --- | --- | --- | --- | --- | --- | --- | --- |
| **Gray matter** | Moran  et al | Li  et al | Chen  et al | Natalia  et al | Wang  et al | Yoon  et al | Zhang  et al | Zhou  et al | Wei  et al | Duan  et al | Feng  et al |
| **Participants、intervention、comparison** |  |  |  |  |  |  |  |  |  |  |  |
| ★Patients were evaluated prospectively, specific diagnostic criteria were applied, and demographic data were reported (0 or 1) | 1 | 1 | 1 | 1 | 1 | 1 | 1 | 1 | 1 | 1 | 1 |
| ★Healthy comparison participants were evaluated prospectively; psychiatric and medical illnesses were excluded (0 or 1) | 1 | 1 | 1 | 1 | 1 | 1 | 1 | 1 | 1 | 1 | 1 |
| ★Important variables (e.g., age, gender, drug status, illness duration, and BMI) were checked either via stratification or statistics (1 or 0.5) | 0.5 | 1 | 0.5 | 0.5 | 1 | 1 | 1 | 1 | 1 | 1 | 1 |
| ★All patients were comorbidity free (0 or 1) | 1 | 1 | 1 | 1 | 1 | 1 | 1 | 1 | 1 | 1 | 1 |
| ★All patients were medication naïve (0 or 1) | 0 | 0 | 0 | 0 | 0 | 0 | 1 | 0 | 0 | 0 | 0 |
| ★Sample size per group: ≥ 10; ≥ 20 (1 or 0.5) | 1 | 1 | 0.5 | 1 | 1 | 1 | 1 | 0.5 | 1 | 1 | 1 |
| **Neuroimaging method and analysis** |  |  |  |  |  |  |  |  |  |  |  |
| ★Magnet strength: 3T; 1.5T (1 or 0.5) | 0.5 | 1 | 1 | 1 | 1 | 0.5 | 1 | 0.5 | 0.5 | 1 | 1 |
| ★The imaging technique used was clearly described so that it could be reproduced (0.5 or 1) | 1 | 1 | 0.5 | 1 | 1 | 0.5 | 1 | 0.5 | 0.5 | 0.5 | 1 |
| ★Whole brain analysis was automated without a previously defined region (0 or 1) | 1 | 1 | 1 | 1 | 1 | 1 | 1 | 1 | 1 | 1 | 1 |
| ★Spatial coordinates were reported in a standard space (e.g., Talairach or MNI coordinates) (0.5 or 1) | 0.5 | 1 | 1 | 1 | 1 | 1 | 1 | 1 | 1 | 1 | 1 |
| **Outcomes and conclusion** |  |  |  |  |  |  |  |  |  |  |  |
| ★Statistical results were corrected for multiple comparison ; uncorrected (1 or 0.5) | 1 | 1 | 1 | 0.5 | 0.5 | 1 | 1 | 0.5 | 0.5 | 1 | 1 |
| ★Conclusions were consistent with the results obtained, and the limitations were discussed (0 or 1) | 1 | 1 | 1 | 0.5 | 0.5 | 1 | 1 | 0.5 | 0.5 | 1 | 1 |
| **Total score** | 9.5 | 11 | 9.5 | 9.5 | 10 | 10 | 11.5 | 8 | 9 | 10.5 | 11 |

| **White matter** | Cui  et al | Kim  et al | Xiong  et al | Yau  et al | Yoon  et al |
| --- | --- | --- | --- | --- | --- |
| **Participants、intervention、comparison** |  |  |  |  |  |
| ★Patients were evaluated prospectively, specific diagnostic criteria were applied, and demographic data were reported (0 or 1) | 1 | 1 | 1 | 1 | 1 |
| ★Healthy comparison participants were evaluated prospectively; psychiatric and medical illnesses were excluded (0 or 1) | 1 | 1 | 1 | 1 | 1 |
| ★Important variables (e.g., age, gender, drug status, illness duration, and BMI) were checked either via stratification or statistics (1 or 0.5) | 1 | 1 | 1 | 1 | 1 |
| ★All patients were comorbidity free (0 or 1) | 1 | 1 | 1 | 1 | 1 |
| ★All patients were medication naïve (0 or 1) | 0 | 0 | 0 | 1 | 0 |
| ★Sample size per group: ≥ 10; ≥ 20 (1 or 0.5) | 1 | 1 | 1 | 0.5 | 1 |
| **Neuroimaging method and analysis** |  |  |  |  |  |
| ★Magnet strength: 3T; 1.5T (1 or 0.5) | 1 | 1 | 1 | 0.5 | 0.5 |
| ★The imaging technique used was clearly described so that it could be reproduced (0.5 or 1) | 1 | 1 | 1 | 1 | 0.5 |
| ★Whole brain analysis was automated without a previously defined region (0 or 1) | 1 | 1 | 1 | 1 | 1 |
| ★Spatial coordinates were reported in a standard space (e.g., Talairach or MNI coordinates) (0.5 or 1) | 1 | 1 | 1 | 1 | 1 |
| **Outcomes and conclusion** |  |  |  |  |  |
| ★Statistical results were corrected for multiple comparison ; uncorrected (1 or 0.5) | 1 | 1 | 1 | 1 | 1 |
| ★Conclusions were consistent with the results obtained, and the limitations were discussed (0 or 1) | 1 | 1 | 1 | 1 | 1 |
| **Total score** | 11 | 11 | 11 | 11 | 10 |

**Table 5. The significant cognitive exanimation information of total 15 studies included**

| **Gray matter** | **Tools of cognition assessment** | **Result (mean ± sd.)** | | **P value** |
| --- | --- | --- | --- | --- |
|  |  | **PT** | **HC** |  |
| Moran et al | Hopkins immediate | 23.7 (5.6) | 21.8 (6.6) | < 0.001 |
|  | RCFT copy | 28.1 (6.5) | 31.6 (6.0) | < 0.001 |
|  | RCFT delay | 12.7 (6.5) | 14.6 (7.1) | < 0.001 |
|  | Symbol search | 24.5 (7.6) | 22.5 (8.0) | < 0.001 |
|  | COWAT category | 18.4 (4.8) | 17.0 (5.1) | < 0.001 |
| Li et al | MMSE | 27.8 (0.2) | 28.5 (0.2) | 0.003 |
|  | MoCA | 22.1 (0.4) | 27.7 (0.2) | <0.0001 |
| Chen et al☆ | MMSE | 25.5 (1.7) | NA | NA |
| Natalia et al | MMSE | 28.8 (1.3) | 29.7 (0.7) | 0.002 |
|  | HDRS | 11.9 (8.9) | 3.2 (3.4) | 0.0001 |
|  | Digit-Symbol test | 34.1 (12.1) | 42.2 (12.2) | 0.02 |
|  | Text Recall (WMS-III) | 8.7 (4.2) | 14.7 (3.0) | 0.0001 |
|  | ROCF copy | 33.8 (2.3) | 35.2 (0.9) | 0.004 |
|  | ROCF delayed memory | 18.4 (5.8) | 22.7 (5.0) | 0.03 |
|  | Trail-making test B | 119.3 (66.3) | 74.5 (26.6) | 0.004 |
|  | STROOP Color-Word | 33.0 (11.1) | 41.3 (9.4) | 0.005 |
|  | Semantic Fluency | 19.2 (6.1) | 24.2 (4.5) | 0.006 |
| Wang et al Δ | MoCA | 23.0 (17–28) | 27 (19–30) | 0.007 |
|  | Clock drawing test | 2.0 (1–4) | 4 (1–4) | 0.002 |
| Yoon et al★ | —— | —— | —— | —— |
| Zhang et al | MoCA | 27.48 (1.33) | 16.43 (3.38) | 0.000 |
| Zhou et al | VLT - delay recall | 5.7 (1.8) | 7.3 (1.4) | < 0.01 |
|  | AVLT - recognition | 20.8 (2.6) | 22.7 (1.2) | < 0.05 |
|  | Clock drawing test | 8.0 (1.1) | 9.5 (2.5) | < 0.05 |
| Wei et al | MMSE | 26.52 (2.05) | 27.59 (11.90) | 0.034 |
| Duan et al | MoCA | 22.50 (2.94) | 24.15 (2.65) | < 0.05 |
|  | Digit span test (DST) | 8.96 (1.48) | 10.08 (1.27) | < 0.05 |
|  | Trail-making test, TMT-B | 149.35 (57.69) | 123.45 (34.37) | < 0.05 |
| Feng et al | GPT (R) Δ | 67.5 (60.25, 71) | 60 (56, 65.75) | 0.045 |
|  | SDT | 52.8 (10.15) | 61.9 (10.29) | 0.008 |

| **White matter** | **Tools of cognition assessment** | **Result (mean ± sd.)** | | **P value** |
| --- | --- | --- | --- | --- |
|  |  | **PT** | **HC** |  |
| Cui et al | CFT-delay | 34.0 (2.6) | 35.0 (1.5) | 0.001 |
|  | TMT-part B | 167.1 (47.7) | 149.4 (51.3) | 0.01 |
| Kim et al * | —— | —— | —— | —— |
| Xiong et al | MoCA | 25.25 (1.25) | 28.64 (1.22) | < 0.001 |
|  | MMSE | 25.40 (2.09) | 28.36 (0.99) | < 0.001 |
| Natalia et al | Emotional parag. – imm | 25.56 (5.75) | 29.53 (6.08) | 0.04 |
| Yoon et al★ | —— | —— | —— | —— |

**☆:** According the diagnosis standard [1]; ★**:** Showed with statistic graphs; **Δ:** Use the median**.**

*: Discussed in study; ★**:** Showed with statistic graphs

[1] Arevalo-Rodriguez I, Smailagic N, Roqué I Figuls M, Ciapponi A, Sanchez-Perez E, Giannakou A, Pedraza OL, Bonfill Cosp X, Cullum S. Mini-Mental State Examination (MMSE) for the detection of Alzheimer's disease and other dementias in people with mild cognitive impairment (MCI). Cochrane Database Syst Rev. 2015 Mar 5;2015(3):CD010783. doi: 10.1002/14651858.CD010783.pub2.

| **Table 6. Heterogeneity analysis of main results in GM/WM meta-analysis** | | | | | | |
| --- | --- | --- | --- | --- | --- | --- |
| 1. Template: *gray_matter*. Anisotropy: *1.0*. Isotropic FWHM: *20mm*. Mask: *gray_matter* | | | | | | |
| **Gray matter** | | **Maximum** | |  | **Minimum** | |
|  |  | **MNI** | **Region** |  | **MNI** | **Region** |
| Li | Whole volume | (none) | |  | -42, -22,20 | Left rolandic operculum, BA 48 |
|  | Mask | (none) | |  | -42, -22,20 | Left rolandic operculum, BA 48 |
| Natalia | Whole volume | (none) | |  | -14,16, -22 | Left superior frontal gyrus, orbital part, BA 11 |
|  | Mask | (none) | |  | -14,16, -22 | Left superior frontal gyrus, orbital part, BA 11 |
| Zhang | Whole volume | (none) | |  | -32, -70, -12 | Left inferior network, inferior longitudinal fasciculus |
|  | Mask | (none) | |  | -32, -70, -12 | Left inferior network, inferior longitudinal fasciculus |
| Chen | Whole volume | (none) | |  | 34,22,40 | Corpus callosum |
|  | Mask | (none) | |  | 34,22,40 | Corpus callosum |
| Moran | Whole volume | (none) | |  | (none) | |
|  | Mask | (none) | |  | (none) | |
| Yoon | Whole volume | (none) | |  | -58, -52, -30 | (undefined), BA 20 |
|  | Mask | (none) | |  | -58, -52, -30 | (undefined), BA 20 |
| Wang | Whole volume | (none) | |  | 62, -38,12 | Right superior temporal gyrus, BA 42 |
|  | Mask | (none) | |  | 62, -38,12 | Right superior temporal gyrus, BA 42 |
| Zhou | Whole volume | (none) | |  | -34,36,32 | Left middle frontal gyrus, BA 46 |
|  | Mask | (none) | |  | -34,36,32 | Left middle frontal gyrus, BA 46 |
| Duan | Whole volume | (none) | |  | 4,36, -14 | Right superior frontal gyrus, medial orbital, BA 11 |
|  | Mask | (none) | |  | 4,36, -14 | Right superior frontal gyrus, medial orbital, BA 11 |
| Wei | Whole volume | (none) | |  | -54, -20, -22 | Left middle temporal gyrus, BA 20 |
|  | Mask | (none) | |  | -54, -20, -22 | Left middle temporal gyrus, BA 20 |
| Feng | Whole volume | (none) | |  | 12,68,4 | Right superior frontal gyrus, medial, BA 10 |
|  | Mask | (none) | |  | 12,68,4 | Right superior frontal gyrus, medial, BA 10 |

| 2) Negative peaks (τ = 0.429, Q = 46.682, df = 9, P < 0.000) | | | | | | |
| --- | --- | --- | --- | --- | --- | --- |
| **Gray matter** | ***d*** | **SE** | ***z*** | ***P*** | ***CI*_low_** | ***CI*_up_** |
| Li | -1.149 | 0.290 | -3.963 | 0.000073996 | -1.717 | -0.581 |
| Natalia | -1.310 | 0.314 | -4.173 | 0.000030016 | -1.925 | -0.695 |
| Zhang | -1.094 | 0.285 | -3.834 | 0.000125865 | -1.654 | -0.535 |
| Chen | -5.954 | 1.074 | -5.546 | 0.000000029 | -8.058 | -3.850 |
| Yoon | -0.497 | 0.176 | -2.830 | 0.004651469 | -0.841 | -0.153 |
| Wang | -1.351 | 0.329 | -4.101 | 0.000041108 | -1.997 | -0.706 |
| Zhou | -1.479 | 0.361 | -4.095 | 0.000042217 | -2.187 | -0.771 |
| Duan | -0.323 | 0.197 | -1.637 | 0.101690046 | -0.710 | 0.064 |
| Wei | -0.911 | 0.266 | -3.431 | 0.000601780 | -1.431 | -0.391 |
| Feng | -1.572 | 0.366 | -4.293 | 0.000017659 | -2.289 | -0.854 |

| 1. Template: fractional_anisotropy. Anisotropy: 1.0. Isotropic FWHM: 20mm. Mask: white_matter | | | | | | |
| --- | --- | --- | --- | --- | --- | --- |
| **White matter** | | **Maximum** | |  | **Minimum** | |
|  |  | **MNI** | **Region** |  | **MNI** | **Region** |
| Cui | Whole volume | (none) | |  | 6, -88,4 | Right calcarine fissure / surrounding cortex, BA 17 |
|  | Mask | (none) | |  | 6, -88,4 | Right calcarine fissure / surrounding cortex, BA 17 |
| Kim | Whole volume | (none) | |  | -28, -64,18 | Corpus callosum |
|  | Mask | (none) | |  | -28, -64,18 | Corpus callosum |
| Xiong | Whole volume | (none) | |  | (none) | |
|  | Mask | (none) | |  | (none) | |
| Yau | Whole volume | (none) | |  | -30,2, -22 | Left amygdala, BA 34 |
|  | Mask | (none) | |  | -40, -22, -18 | Left inferior network, inferior longitudinal fasciculus |
| Yoon | Whole volume | (none) | |  | 22, -86,4 | Right inferior network, inferior longitudinal fasciculus |
|  | Mask | (none) | |  | 22, -86,4 | Right inferior network, inferior longitudinal fasciculus |

| 4) Negative peaks (τ = 0.084, Q = 7.056, df = 3, P > 0.05) | | | | | | |
| --- | --- | --- | --- | --- | --- | --- |
| **White matter** | ***d*** | **SE** | ***z*** | ***P*** | ***CI*_low_** | ***CI*_up_** |
| Cui | -0.789 | 0.233 | -3.390 | 0.000698456 | -1.246 | -0.333 |
| Kim | -0.949 | 0.335 | -2.830 | 0.004648167 | -1.606 | -0.292 |
| Yau | -1.090 | 0.341 | -3.193 | 0.001406759 | -1.758 | -0.421 |
| Yoon | -0.286 | 0.174 | -1.644 | 0.100191973 | -0.627 | 0.055 |

| **Table 7. Jackknife analysis of main results in GM/WM meta-analysis** | | | | | | | | | | | | |
| --- | --- | --- | --- | --- | --- | --- | --- | --- | --- | --- | --- | --- |
| 1. **Jackknife analysis results of GM (T2DM-CD VS. HCs)** | | | | | | | | | | | | |
| **GM** | **Chen** | **Duan** | **Feng** | **Li** | **Moran** | **Natalia** | **Wang** | **Wei** | **Yoon** | **Zhang** | **Zhou** | **Total** |
| Right superior temporal gyrus, BA 21 | Y | Y | Y | N | N | Y | Y | Y | N | N | Y | 7/11 |
| Right superior frontal gyrus, medial orbital, BA 11 | Y | Y | Y | Y | Y | N | Y | Y | Y | Y | Y | 10/11 |
| Left superior temporal gyrus, BA 48 | Y | Y | Y | N | Y | Y | Y | Y | N | Y | N | 8/11 |
| Left middle occipital gyrus, BA 19 | Y | Y | Y | N | N | Y | N | N | Y | N | Y | 6/11 |
| Left inferior network, inferior fronto-occipital fasciculus | Y | Y | N | N | N | N | Y | Y | Y | Y | Y | 7/11 |
| Left middle temporal gyrus, BA 21 | N | Y | Y | Y | N | N | Y | N | Y | N | N | 5/11 |
| Left median cingulate / paracingulate gyri, BA 23 | N | Y | Y | Y | N | N | Y | Y | Y | N | Y | 7/11 |
| Right calcarine fissure / surrounding cortex, BA 17 | Y | Y | Y | N | Y | Y | Y | Y | N | Y | N | 8/11 |
| Left fusiform gyrus, BA 19 | Y | Y | Y | Y | N | N | N | Y | Y | N | N | 6/11 |
| Right middle frontal gyrus, BA 9 | N | Y | Y | Y | N | N | Y | Y | Y | Y | N | 7/11 |

| 1. **Jackknife analysis results of WM (T2DM-CD VS. HCs)** | | | | | | |
| --- | --- | --- | --- | --- | --- | --- |
| **WM** | **Cui** | **Kim** | **Xiong** | **Yau** | **Yoon** | **Total** |
| Corpus callosum (18, -36,30) | Y | N | N | Y | N | 2/5 |
| Corpus callosum (-26, -70,16) | Y | N | N | N | N | 1/5 |
| Left inferior network, uncinate fasciculus | Y | Y | Y | N | N | 3/5 |
| undefined (10, -86,6) | N | Y | Y | Y | N | 3/5 |
| Left calcarine fissure / surrounding cortex, BA 17 | N | N | N | N | Y | 1/5 |
| Right inferior network, inferior fronto-occipital fasciculus | Y | Y | Y | Y | N | 4/5 |
| Corpus callosum (-20,36,8) | Y | Y | N | Y | N | 3/5 |
| undefined (-34, -54, -44) | N | Y | Y | Y | N | 3/5 |
| Right inferior network, inferior longitudinal fasciculus | Y | Y | Y | Y | N | 4/5 |
| Right cortico-spinal projections | N | Y | Y | Y | N | 3/5 |
| undefined (32, -60, -42) | Y | Y | Y | Y | N | 4/5 |


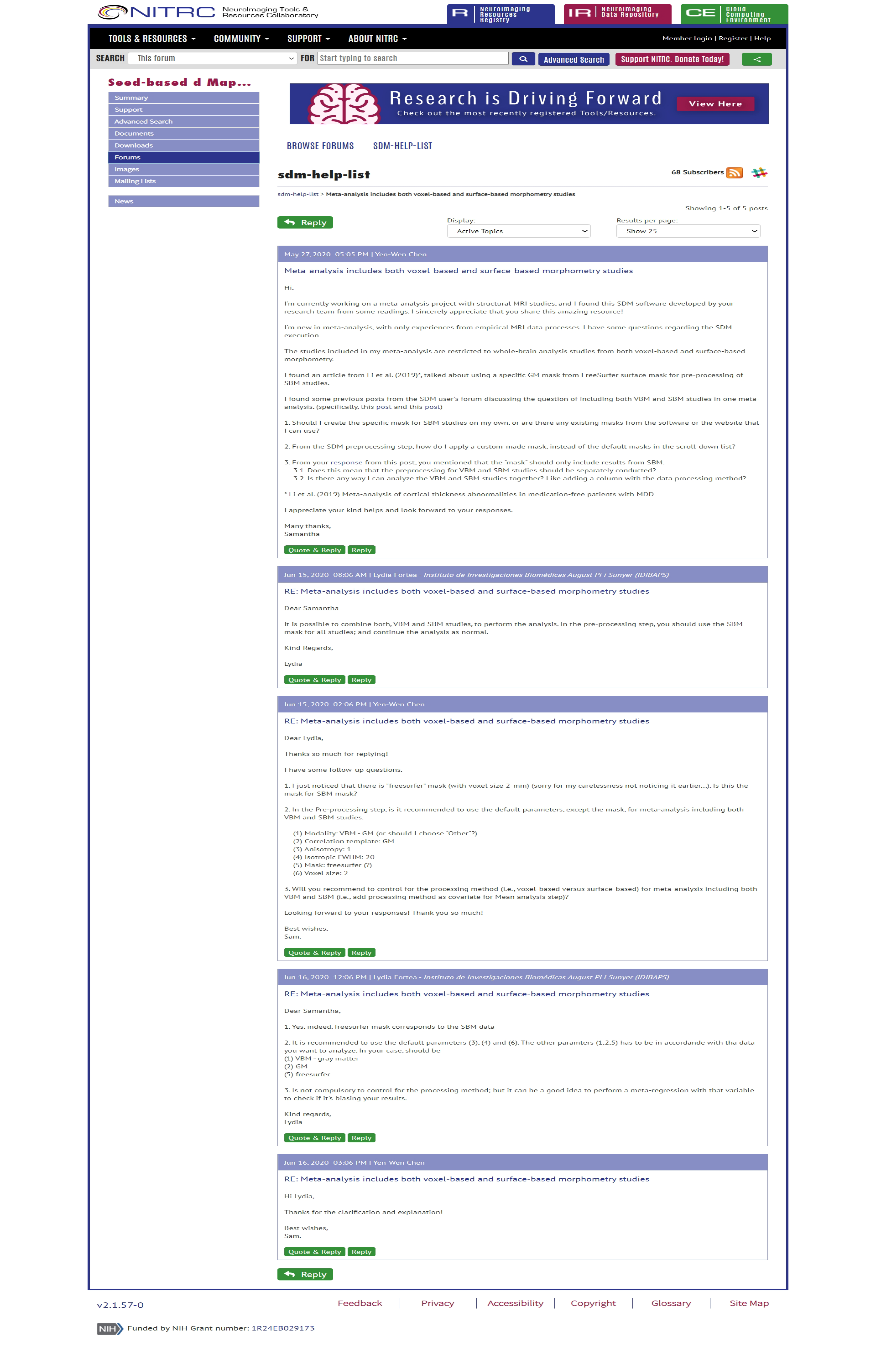


**Fig. 1 The approach to pool the VBM and SBM studies into meta-analysis**


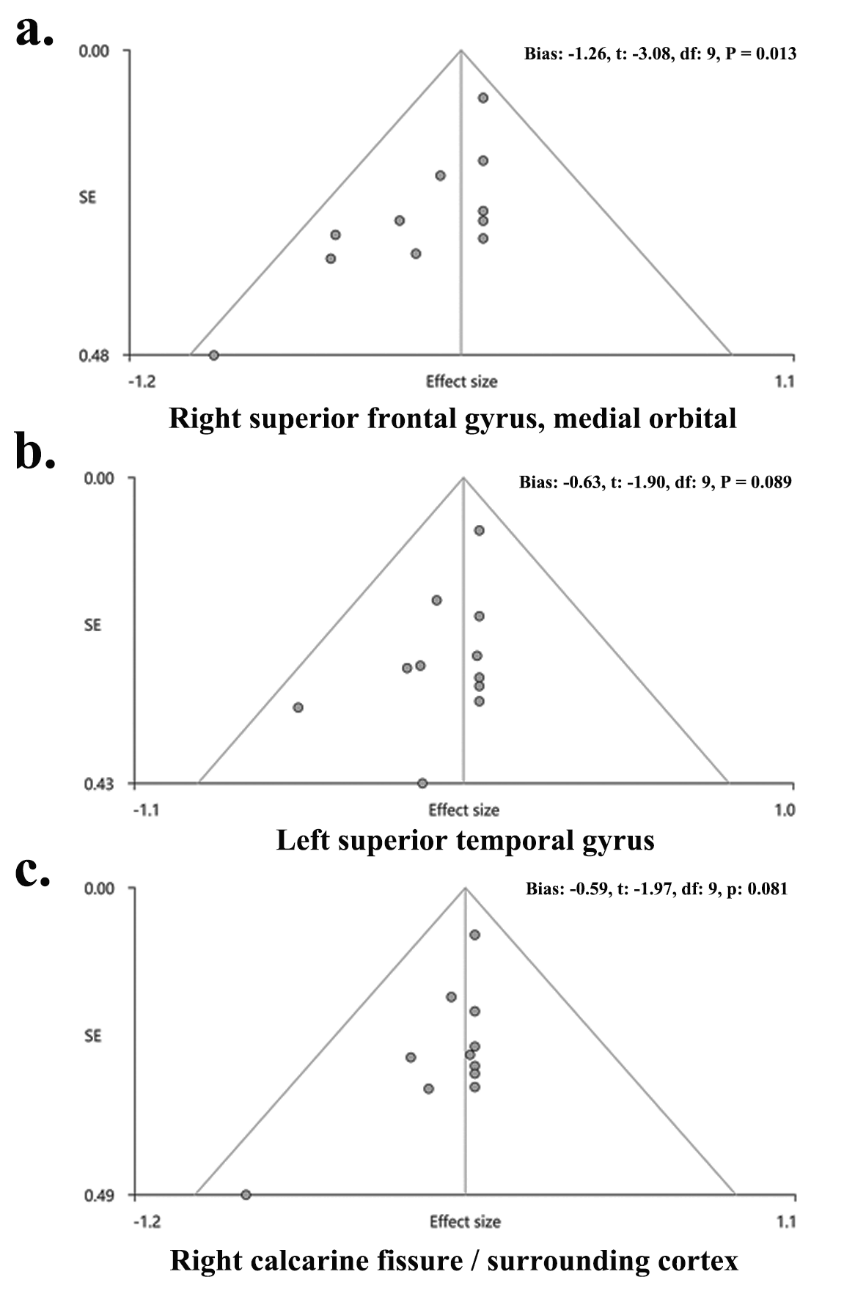


**Fig. 2: Funnel plot of the robust main results in GM studies**

**
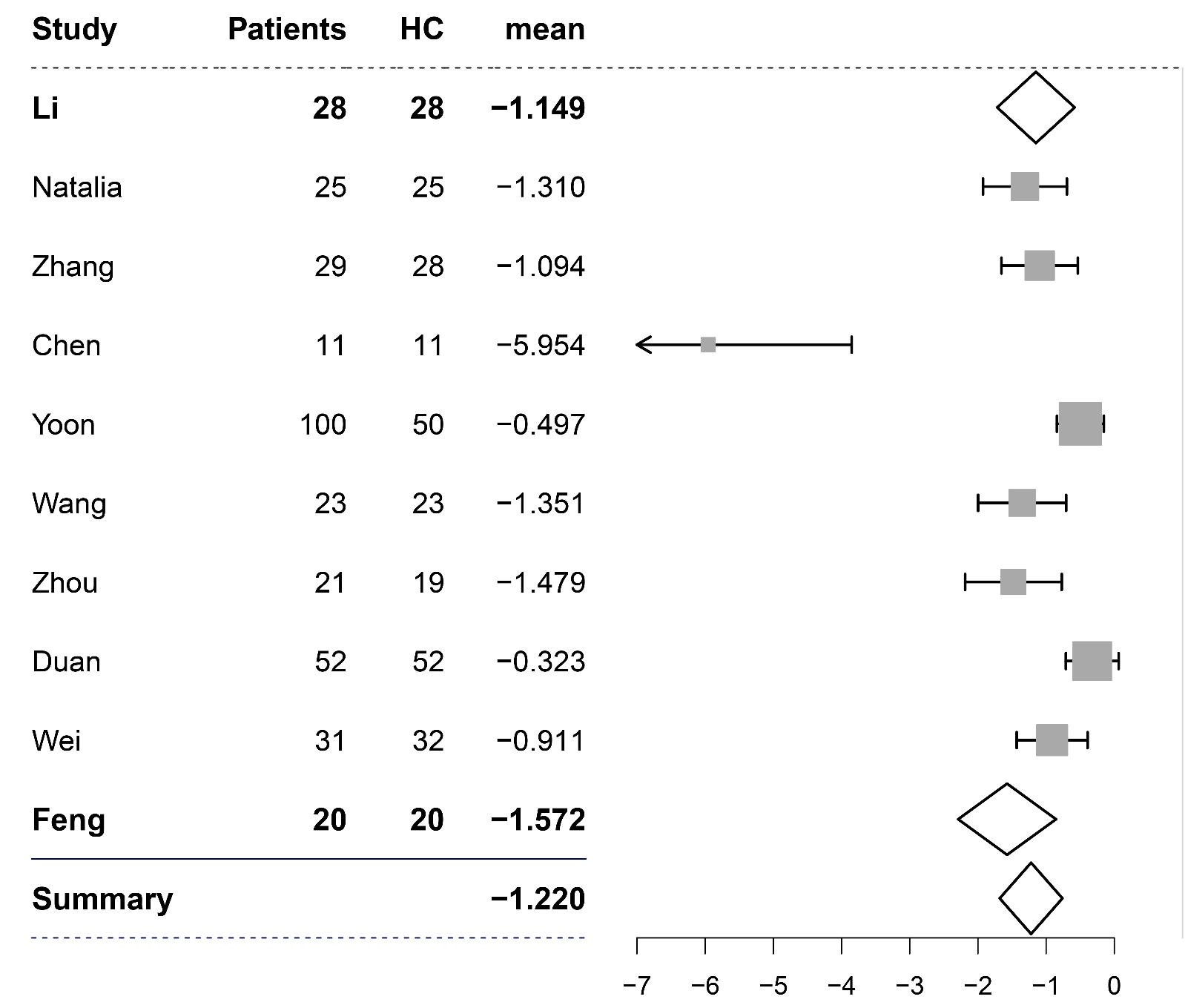
**

**Fig. 3: Forest plot of the heterogeneity analysis in GM studies
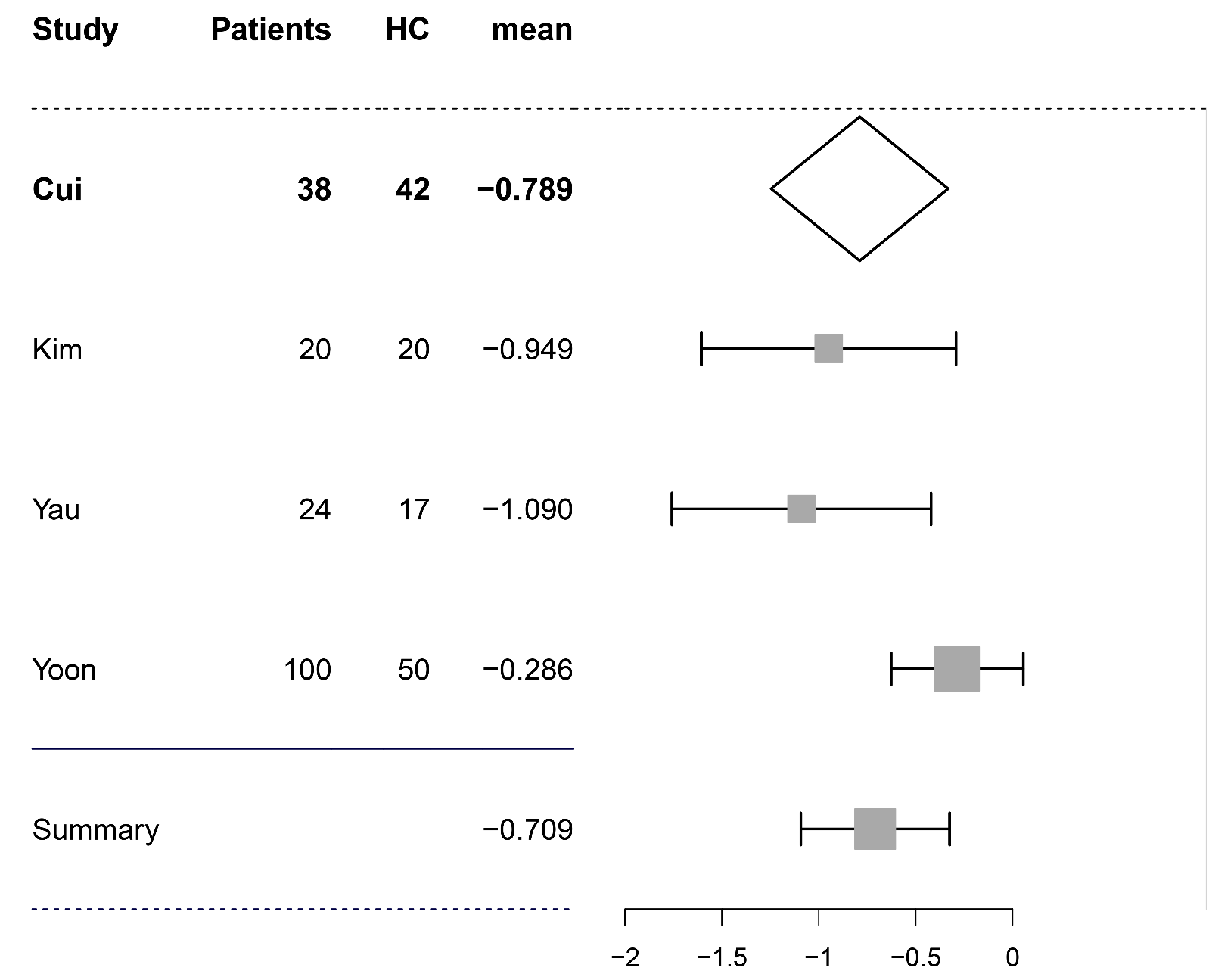
**

**Fig. 4: Forest plot of the heterogeneity analysis in WM studies**

**Ethical statement**

**Conflict of Interest:** The authors declare that the research was conducted in the absence of any commercial or financial relationships that could be construed as a potential conflict of interest.

**Contribution statement:** GBC, LFY and WW made the article framework design; GBC and LFY provided the funding support; TM, ZYL and LFY performed the systemic search, study selection and data analysis; YY, BH, YH, MHN, YXH and HHC checked the methods and results, searched the reference martials, and contributed to the interpretation of findings; TM, ZYL and LFY wrote the original draft; GBC and WW revise the article.

**Fundings:** This study was supported by the National Natural Science Foundation of China (No.81771815, GBC) and the Military Medical Enhancement Program of Air Force Medical University (No.2018HKPY03, GBC; No.2018JSTS13, LFY).

**Data access:** The coordinate information and their original studies were publicly available.

**Policy and ethics:** All studies included in this meta-analysis were published.

**Originality and plagiarism:** The authors made sure to write completely original work. The work and/or words of others were appropriately cited or quoted.
